# Preparing for parenthood: molecular reprogramming precedes parenting in the burying beetle *Nicrophorus vespilloides*

**DOI:** 10.1186/s12983-026-00615-4

**Published:** 2026-05-18

**Authors:** Julius Rombach, Heiko Vogel, Sandra Steiger, Volker Nehring

**Affiliations:** 1 Department for Evolutionary Biology and Ecology, Institute of Biology I (Zoology), University of Freiburg, Hauptstraße 1, 79104 Freiburg im Breisgau, Germany; 2 Department of Insect Symbiosis, Max-Planck-Institute for Chemical Ecology, Hans-Knöll-Straße 8, 07745 Jena, Germany; 3 Department of Evolutionary Animal Ecology, University of Bayreuth, 95447 Bayreuth, Germany

**Keywords:** Juvenile hormone, Life history, Offspring provisioning, Parental care, Social evolution, Social immunity, Transcriptional regulation, Vitellogenin

## Abstract

**Background:**

Subsocial insects transition from solitary to social behavior during reproduction. They engage in cooperative and parental care activities that increase their offspring’s survival. Elucidating the molecular mechanisms underlying such social transitions is crucial for understanding the genetic basis of social behavior. Burying beetles reproduce on a nutritious yet ephemeral resource. They provide intensive pre-hatching care by burying and preparing a vertebrate carcass, and post-hatching care by feeding the larvae.

**Results:**

We analyzed female gene expression during the transition from solitary life to brood care in the subsocial burying beetle Nicrophorus vespilloides. We collected RNA-seq data from the heads and fat bodies of beetles at four different time points: solitary life, early pre-hatching care, late pre-hatching care, and post-hatching care. The most pronounced shift in gene expression occurred during the initial onset of social behavior. Carcass discovery caused massive changes in the transcription of genes involved in metabolism, translation, and immunity. Changes in gene expression between the different brood care phases were comparatively modest: Females upregulated the expression of genes associated with social immunity as parental care intensified. During brood care, they gradually downregulated fecundity-related genes.

**Conclusions:**

The regulatory pattern indicates that carcass discovery triggers an increased metabolism to cope with the demands of brood care, alongside a reduced investment in body maintenance. The overall regulatory pattern indicates a strong investment into the current reproduction bout at the expense of future reproduction.

**Supplementary Information:**

The online version contains supplementary material available at 10.1186/s12983-026-00615-4.

## Background

Social behavior ranges from simple, transient interactions between individuals to complex societies. Subsocial species live in temporary parent-offspring associations and engage in parental care [1, 2]. Subsociality has arisen independently in at least 13 different orders of insects [3, 4] and is believed to be a primary evolutionary route to complex forms of sociality [1]. In recent years, many studies shed light on the genetic basis of social behavior, especially in eusocial Hymenoptera [5–8]. Early stages of sociality are associated with regulatory changes that alter the timing and patterns of gene expression. These changes in gene expression may lead to behaviors where individuals cooperate in tasks such as brood care or nest defense [9, 10] and often target conserved pathways [11]. Gene regulation involved in social behavior appears to be shared across lineages and levels of sociality with similar regulatory pathways co-opted from ancestral functions [5, 12].

Juvenile hormone (JH) stands out as a conserved pleiotropic regulator of both reproductive and behavioral phenotypes, with a role in modulating social behaviors from subsocial to eusocial species. It plays an important role as mediator of development and affects the transitions between different life stages [13]. In eusocial insects, JH can determine whether an individual develops into a reproductive or a sterile caste [14]. JH has also been linked to the regulation of worker tasks such as nursing, foraging, and nest colony defense [14, 15]. For instance, in some eusocial insects, queens produce pheromones that modulate worker behavior and development by influencing worker JH levels, thereby regulating the social structure of the colony [16, 17]. In subsocial insects, development and temporary parental care are also linked to JH, however patterns of JH regulation vary [18, 19].

The subsocial burying beetles reproduce on small vertebrate carcasses [20, 21]. A sharp rise in JH levels occurs upon carcass discovery [22] and again following the appearance of larvae on the carcass. This second peak happens during the post-hatching care phase, after which JH levels gradually decline. The timing coincides with reduced fertility of females and intense larval feeding, suggesting JH levels could induce post-hatching parental care [22–28]. Further evidence for this comes from the observation that the second peak is exclusive to brood-caring burying beetles and absent in the close relative *Ptomascopus morio*, where parents do not directly interact with their offspring [29, 30].

JH titers are regulated through a set of enzymes that synthesize or degrade JH. The JH biosynthesis in the *corpora allata* (CA) is partially regulated by the expression of JH acid O-methyltransferase (*jhamt*) and methyl farnesoate epoxidase (*cyp15a1*) genes, while degradation depends on the expression of JH esterase (*jhe*) and JH epoxide hydrolase (*jheh*), which inactivate JH [31, 32]. Components of the IIS-ToR pathway such as InR (insulin receptor), Protein kinase B (Akt), and Forkhead-Box-O (FOXO) regulate JH biosynthesis based on nutritional cues [32]. Downstream, high JH titres typically cause high *Krüppel homolog (Kr-h1*) expression, a transcription factor that mediates JH-responsive gene expression and that also feeds back on genes controlling JH synthesis and degradation [33].

Besides its role in development, JH is involved in regulating fecundity, for example by controlling the expression of *vitellogenin* (*vg*), which codes for a storage protein that plays a central role in insect egg production [34]. However, it is hypothesized that, beyond its established role in reproduction, Vg may serve a regulatory function as a signal for behavioral change. In the honey bee, higher JH titers in foragers are associated with reduced *vg* gene expression [35], a pattern also seen in burying beetles during the transition to parental care [23, 36–39]. In female burying beetles, reduced *vg* expression is associated with a temporary suppression of reproduction and shift towards active care for offspring [38]. However, the regulatory mechanism of *vg* expression in burying beetles remains unclear.

Social behavior in insects is also controlled by neuromodulators such as dopamine [37, 40, 41]. In the burying beetle *Nicrophorus orbicollis*, dopamine levels increase during larval care compared to non-breeding females, suggesting a role in facilitating parental behavior [42]. Neuropeptide F (NPF) and its receptor, homologous to vertebrate neuropeptide Y signaling, are associated with feeding motivation in insects [43, 44]. During social evolution, such neuromodulatory pathways may have been co-opted to regulate behaviors like cooperation and brood care. For example, in honey bees, *npf* expression is lower in foraging compared to nursing workers, suggesting a role in caregiving [45]. In *Nicrophorus vespilloides*, reduced *npf-receptor* expression during post-hatching suggests a shift in motivation from self-feeding toward feeding offspring which is a key component of burying beetle parental care [46].

Other gene families may also contribute to the regulation of social behaviors. Takeout proteins, part of the Takeout/juvenile hormone-binding protein (JHBP) family, are implicated in regulating feeding behavior, circadian rhythms, and potentially social interactions in insects [47, 48]. Several takeout-like genes have been identified in the transcriptomes of burying beetles. In *N. vespilloides* females, *takeout* was significantly downregulated during post-hatching care compared to the pre- and post-caring stages in the head [37]. Notably, recent work on *N. orbicollis* suggests that a specific *takeout* may be involved in temporal kin recognition, with increased gene expression observed in the heads of females that accepted larvae compared to those that rejected them [49].

Social immunity is a key innovation in the evolution of sociality [50]. By reducing pathogen pressure within a group, immune strategies help to protect group members and shared resources [50]. Social immunity can be mediated through behavioral adaptations as well as through physiological mechanisms such as the external use of immune effectors [51]. Among the most important external immune effectors are antimicrobial peptides (AMPs), which, together with peptidoglycan recognition proteins and lysozymes, are part of insect innate immunity. While these molecules play a central role in defending individuals internally against microbial infection, they can also function at the group level by limiting pathogen growth in the shared environment. In burying beetles, reproduction depends on small vertebrate carcasses, which are highly nutritious but naturally prone to rapid microbial and fungal degradation. Adults apply oral and anal secretions containing external immune effectors that suppress microbial activity, to prevent the accumulation of toxic substances and to preserve carcass quality [52]. In doing so, they indirectly enhance offspring survival [53, 54]. Interestingly, the AMP and lysozyme levels detected in the anal secretions of *N. vespilloides* have been shown to vary across parental care phases, suggesting that specific peptides are functionally evolved for social immunity [55].

**Fig. 1:**
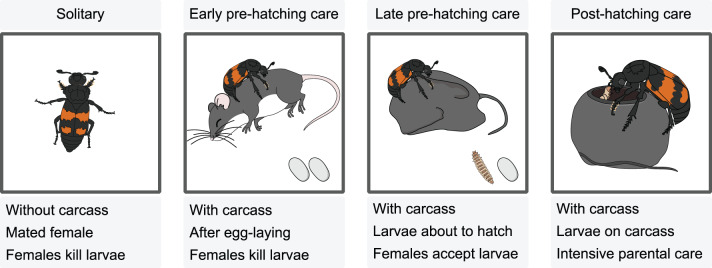
Experimental setup. Head and fat body RNA was collected from female beetles during a solitary phase and three parental care phases

Despite growing insight into the molecular underpinnings of social behavior, the genetic regulation of parental care in early social evolution remains poorly understood. Here, we examine the molecular regulation of brood care in the subsocial burying beetle *N. vespilloides*. Burying beetles bury carcasses to defend them from vertebrate and invertebrate competitors. They also “embalm” them with antimicrobial oral and anal secretions before and during egg laying (pre-hatching care, Fig. 1 [20, 21]). Beetles feed on the carcass and gain weight, suggesting that they accumulate energy reserves in anticipation of costly reproductive activities [56–58]. However, as the pre-hatching phase progresses, weight gain declines, likely reflecting increased energy expenditure on caregiving tasks [57]. The parents are infanticidal during early pre-hatching care to make sure they do not invest into the offspring of parasitic females, however females do start to accept larvae on the carcass just before their own offspring hatch [59]. When larvae aggregate on the carcass, they beg for food and are fed by parents through regurgitations of pre-digested carrion, which increases offspring fitness [60]. As a result of the ephemeral nature of the resource, eggs develop rapidly, leaving the beetles with only a short window to prepare for feeding their offspring. [61, 62]). Beetles must thus quickly adjust their physiology to brood care, to ensure offspring survival.

We analyzed gene expression separately in the head and fat body of solitary females and across different phases of brood care to disentangle the molecular regulators and effects underlying pre-hatching care (e.g. carcass preparation) from those associated with direct interactions with offspring (e.g. feeding). We took particular care to separate the physiological changes that lead beetles to accept larvae rather than kill them as potential offspring of brood-parasitic females from those induced by larval presence itself, by examining both the acceptance phase and subsequent interactions with larvae.

## Methods

To identify the central regulators of different components of brood care in burying beetles, we investigated head and fat body gene expression across the behavioral transition from solitary mated females to successive stages of care, from carcass preparation and egg laying to offspring acceptance and active larval feeding (Fig. 1). This temporally fine-grained design allows us to separate the regulation and effects of fecundity and parental care from those caused by the presence of the breeding resource.

### Origin and maintenance of beetles

The burying beetles used for this study were part of an outbred colony of *Nicrophorus vespilloides* (fourth generation). Individuals in this colony were descendants of beetles originally collected in spring 2022 using baited pitfall traps in Bayreuth, Germany. Prior to the experiment, we kept the beetles in plastic containers (10 × 10 × 6 cm) filled with a 3 cm layer of moist soil. Each container included one to three individuals of the same sex and family. Beetles were fed twice a week with thawed green blow fly larvae (*Lucilia sericata,* Hund & Sport Hungenberg GmbH). Containers were stored inside a temperature-regulated climate chamber at 20 °C with a standard photoperiod of 16 : 8 h (light : dark).

### Experimental design

We started our experiment by pairing virgin female and male beetles from different families to prevent sibling mating. To encourage mating, we placed each pair of beetles in an empty plastic container (10 × 10 × 6 cm) and allowed them to interact for one hour. After this time, the beetles were transferred to containers containing a 3 cm layer of moist soil. After 48 hours, we sampled the first batch of females for RNA extraction, which we classified as females in their solitary phase (Fig. 1). This resembles the natural condition, as females are typically mated before they locate a carcass for reproduction. It also ensured that the gene expression changes we measured were not simply caused by mating.

In burying beetles, both biparental and uniparental care naturally occur. To avoid potential variation due to male care we removed the males from the remaining pairs and provided the females with a thawed mouse carcass (weight: 15.5–19.5 g; size: 7–7.5 cm; BAF Petfood GmbH). To replicate natural underground conditions, we kept the females in complete darkness and handled them under red light at a stable temperature of 20 °C for the duration of the experiment. Forty-eight hours after the females received the carcass, we sampled the second batch of females (early pre-hatching care, Fig. 1). Females in the pre-hatching phase had buried the carcass and laid eggs. However, they would not yet accept larvae but kill them instead.

We separated the remaining females from their eggs by transferring the female and the carcass to a new but similar plastic container with moist soil. This allowed us to control the time point at which the females came into contact with larvae and started post-hatching care. Approximately 24 hours later, or as soon as the first larvae hatched for each mother, we sampled the corresponding females, representing the third experimental batch (late pre-hatching care, Fig. 1). These females were classified as being in the late pre-hatching care phase, a phase where the larvae were about to hatch, and the females were primed to accept the presence of any conspecific larvae, but had no contact with larvae at this phase.

We provided the remaining females with a batch of 15 first instar larvae each from their own brood directly on the carcass. If their brood did not produce enough larvae, we added larvae from other females to ensure each female received the same number of larvae. Another 24 hours later, at which time females are expected to reach the peak of intense direct care for larvae [63], we sampled the final batch. Those females were classified as being in their post-hatching care phase (Fig. 1).

### Generation of Transcriptomes

After collecting the females, we immediately dissected heads and fat bodies for RNA extraction (Supplementary Methods). Overall, we collected five head and five fat body samples for each phase, except during the post-hatching care phase, where we obtained only four samples per tissue because of bad RNA quality in one sample.

### RNA-seq data analysis

All downstream data analyses were performed using R Statistical Software 4.4.1 [64] within the RStudio environment [65]. An experimental design file was created to link each sample to its respective tissue and solitary or parental care phase. Head (*N* = 19) and fat body (*N* = 19) samples were then separated for independent analysis. Genes with low expression levels were filtered out by retaining only those genes with a total read count of at least 10 in a minimum of four samples per tissue, corresponding to the smallest group size (unfiltered: 12,642 genes; filtered: head = 11010, fat body = 9393).

### Differential expression analysis

We conducted a differential gene expression analysis using DESeq2 v1.44.0 [66] on the filtered reads with parental care phase as a factor with four levels (Fig. 1). Comparisons were made between successive phases using a Wald test: Early pre-hatching care vs. solitary phase, late pre-hatching care vs. early pre-hatching care and post-hatching care vs. late pre-hatching care (Table S1). We also conducted a DESeq2 analysis using a likelihood ratio test (LRT) by comparing the full model (~ phase) against a reduced model (~1). This approach allowed us to identify genes whose expression levels are significantly influenced by any experimental phase (Table S2). For both approaches genes with an adjusted *p*-value p_*adj*_ < 0.05 using the Benjamini & Hochberg approach were considered as significantly differentially expressed. For several genes, the initial DESeq2 LRT did not return a *p*-value because individual samples were flagged as outliers based on Cook’s distance. For these genes, the affected samples were excluded and the LRT was rerun; the adjusted *p*-values reported here are derived from these reduced datasets.

### Functional enrichment analysis and Gene annotation

To summarize the functions of the differentially expressed genes, we conducted a functional pathway enrichment analysis using Kyoto Encyclopedia of Genes and Genomes (KEGG) terms on pairwise comparisons between successive phases (Supplementary Methods).

We further obtained functional annotations of genes of interest using reference proteomes from different databases with the *N. vespilloides* proteome from NCBI (GCF_001412225.1_Nicve_v1.0) as input. In addition, we used the gene annotations based on the *N. vespilloides* genome on NCBI (Supplementary Methods).

## Results

### Global pattern

**Fig. 2:**
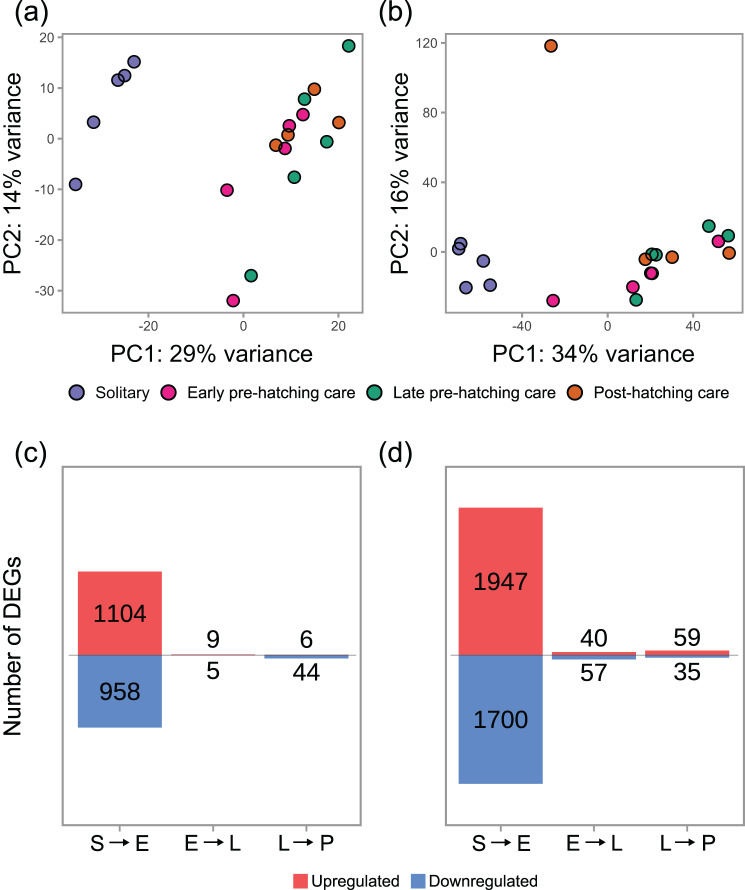
Parental care affected head (**a**) and fat body (**b**) gene expression, with the largest effects caused by the transition onto the carcass. Each dot represents a biological replicate in a principal component analysis based on the expression of all genes, with clustering indicating similarity in expression patterns. The number of genes whose expression was affected by the transitions between subsequent phases (S solitary, E early pre-hatching care, L late pre-hatching care, P post-hatching care) was largest in both head (**c**) and fat body (**d**) when beetles moved onto the reproduction carcass (S → E). Each bar represents the total number of up- (red) and downregulated (blue) genes for each tissue (p_*adj*_ < 0.05)

The largest effect on gene expression in both head and fat body occurred when beetles transitioned from the solitary phase to reproduction upon receiving a carcass (Fig. 2; >2000 differentially expressed genes (DEGs) in both tissues). The number of genes affected by later transitions was an order of magnitude lower in both tissues (Fig. 2c, d). In the fat body, the number of DEGs where similar across transitions within the parental care period (97 and 94 genes; Fig. 1d, Table S1). In the head, the transition from late pre-hatching to post-hatching care phase affected the expression of more genes than the transition from the early to the late pre-hatching care phase (50 vs. 14 genes; Fig. 1c, Table S1).

The transition of beetles onto a carcass was associated with pronounced changes in gene expression across both head and fat body tissues (Fig. S1, Fig. S4; Table S1, Table S4). The most strongly regulated genes in the head included two enzymes involved in fatty acid catabolism (*phytanoyl-CoA dioxygenase-like*, LOC108563537; *gamma-butyrobetaine dioxygenase-like*, LOC108561612), a transcription factor (*TFIIIB component B’’-like*, LOC108562870), and a *CY*P450 enzyme (cytochrome P450 *9e2-like*, LOC108557462) (Fig. S1; Table S1, Table S4). In the fat body, we observed strong regulation of genes putatively involved in cuticular hydrocarbon biosynthesis (*acyl-CoA Delta(11) desaturase-like*, LOC108569014; *fatty acid synthase-like*, LOC108557776) and a fatty acid degradation enzyme (*phytanoyl-CoA dioxygenase-like*, LOC108563537) (Fig. S4; Table S1, Table S4). Additional strongly regulated genes in the fat body included a putative extracellular matrix-binding protein (uncharacterized protein, LOC108562918), a scavenger receptor (*protein croquemort-like*, LOC108565605), and a CYP450 enzyme (*cytochrome P450 9e2-like*, LOC108557462). Among the strongest regulated genes were a digestive protease (*chymotrypsin-1-like*, LOC108566662), two putative odorant-binding proteins (uncharacterized protein LOC108563401, LOC108563402), and two proteins associated with photoreceptor membrane function (uncharacterized protein LOC108559711, LOC108559697) (Fig. S4; Table S1, Table S4).

The transition from the infanticidal to the larval-accepting phase state was likewise accompanied by marked transcriptional changes (Fig. S2, Fig. S5; Table S1, Table S4). In the head, strongly regulated genes included a c-type lysozyme (*clys4*, LOC108566479), a peptidoglycan recognition protein (*PGRP-SC.1*, LOC108561580), and a protein putatively involved in neural regulation (*neo-calmodulin-like*, LOC108559023) (Fig. S2; Table S1, Table S4). In the fat body, prominent responses were observed for a digestion-related enzyme inhibitor (*double-headed protease inhibitor-like, submandibular gland-like*, LOC108560463), a CYP450 enzyme (cytochrome P450 4d2-like, LOC108569000), and a putatively secreted protein (uncharacterized protein LOC108559597) (Fig. S5; Table S1, Table S4). In addition, we detected strong regulation of an antimicrobial peptide attacin-like (uncharacterized protein, LOC108567301), a nutrient storage protein (*hexamerin-like*, LOC108563098), and a digestion-related binding protein (*peritrophin-1-like*, LOC108557847) (Fig. S5; Table S1, Table S4).

**Fig. 3:**
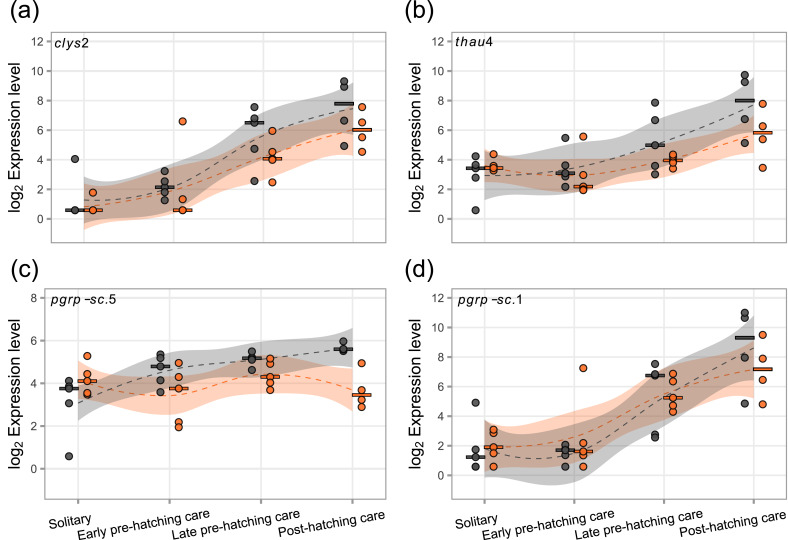
Expression levels of four genes potentially involved social immunity increased from the solitary phase to the post-hatching care phase in the head (grey) and fat body (orange) (**a**: *clys2, c-type lysozyme 2,* LOC108559252; **b**: *thau4 thaumatin 4,* LOC108569648; **d**: *pgrp-sc.1, peptidoglycan recognition protein-sc, LOC108561580;* all LRT *p* < 0.05), with the exception of *pgrp-sc.5*, which did not change in the fat body (**c**: *peptidoglycan recognition protein-sc,* LOC108569068, *p* = 0.71). Dots represent individual samples and dashed lines (LOESS) show expression pattern with shaded areas indicating the 95% confidence interval. Expression levels are plotted as log_2_ transformed normalized counts. Horizontal bar shows median of samples per phase and tissue

When beetles transitioned to caring for larvae the following set of genes showed strong regulation (Fig. 3, Fig. S6; Table S1, Table S4). In the head, this included two digestion-related proteases (*chymotrypsin-2-like*, LOC108562401; *trypsin-1-like*, LOC108557475) and a putative odorant-binding protein (uncharacterized protein, LOC108567616) (Fig. S3; Table S1, Table S4). In the fat body, we again observed regulation of digestion-associated genes, including a protease (*chymotrypsin-1-like*, LOC108559551) and a binding protein (*peritrophin-1-like,* LOC108557847). In contrast, a digestion-related lipase (*gastric triacylglycerol lipase-like,* LOC108558220) was downregulated in both tissues (Fig. S6; Table S1, Table S4).

We further examined uncharacterized genes that exhibited distinct and biologically relevant expression patterns. During the transition from infanticidal to the larval-accepting phase, two uncharacterized genes were upregulated in the fat body. LOC108568756 was found to be orthologous to the *Drosophila melanogaster* gene *geko*, which has been implicated in olfactory responses [67] (Table S1). The second gene, LOC108565117, showed elevated expression both in females anticipating larvae and during active care. A BLASTp hit to the MraZ protein from *Gryllus bimaculatus* suggests a potential role in transcriptional regulation (Table S1). At the onset of larval care, LOC108565455 was upregulated in both tissues, despite stable expression during earlier behavioral phases (LRT *p* < 0.001). Analysis of the conserved protein domain of its *Tribolium castaneum* orthologue indicates a putative function in an ABC-type Mn^2^^+^ /Zn^2^^+^ transport system (Table S1, Table S2).

### Functional enrichment analysis of DEGs using KEGG

To investigate the biological processes associated with the changes across parental care phases, we conducted a functional enrichment analysis using KEGG pathways separately on up- and downregulated genes for each transition between phases, in both head and fat body tissues (Table S3). After *Nicrophorus vespilloides* females arrived at the carcass, pathways related to protein processing, energy production, and basic metabolism were enriched in upregulated genes in both head and fat body tissues (Fig. S7a, S8a; Table S3). We observed some additional tissue-specific patterns as pathways for insect hormone biosynthesis (juvenile hormone biosynthesis) and glycolysis were enriched in upregulated genes in the head (Fig. S7a), while fatty acid metabolism was enriched in the fat body (Fig. S8a). In both tissues, pathways related to ribosome function were enriched in downregulated genes (Fig. S7b, S8b) and TGF-beta signaling in the fat body (Fig. S8b). The later transitions affected fewer genes. Most of these changes affected specific metabolic processes that varied between tissues (Fig. S7c, S8d; Table S3).

### Immune related genes

We detected fewer antimicrobial peptides (AMPs) and lysozymes in the NCBI proteome compared to Jacobs et al. [55], possibly due to some sequences sharing the same top BLAST hit. Overall, 85% of the examined peptidoglycan recognition proteins (PGRPs), AMPs, and lysozymes were expressed, with approximately 60% of these genes displaying differential expression across the parental care phases (Table S2). Most differences in gene expression occurred when beetles transitioned to the carcass. Specifically, expression of a *thaumatin* (*nves-thau4*), a *c-type lysozyme* (*nves-clys2*), and a *PGRP-SC* (*nves-PGRP-SC.1*) increased with the intensification of parental care in both tissues (Fig. 3a, b, d; Table S4), while a second *PGRP-SC* (*nves-PGRP-SC.5*) showed increased expression only in the head (Fig. 3c; Table S4).

### Takeout and neuropeptide F

We found a significant reduction in the expression of the *neuropeptide F receptor* in the head (*nves-npfr*; LOC108564901; Fig. S10b). Expression levels were already significantly lower in the late pre-hatching phase compared to the solitary phase (Wald-p_*adj*_ < 0.01), and declined further during post-hatching care (p_*adj*_ < 0.001; Fig. S10b). The expression of the *short neuropeptide F* (*nves-snpf*; LOC108562743; Fig. S10a) declined in the head; this gene was not expressed in the fat body.

We used reciprocal BLASTp to identify the *Drosophila melanogaster* homologue of *takeout* in *N. vespilloides* (Table S5). In both tissues we found the expression of *takeout* (LOC108560804) to change across phases (Fig. S11). The expression pattern in the head was consistent with the findings of Potticary et al. [49]: *takeout* expression increased significantly during the transition from the solitary to the early pre-hatching care phase (p_*adj*_ < 0.001), and peaked during the late pre-hatching care phase (Fig. S11). In the fat body, *takeout* expression decreased during the transition from the solitary to the early pre-hatching care phase (p_*adj*_ < 0.01, Fig. S11). In both tissues, no significant differences in expression were observed between the early and late pre-hatching care phase.

### Juvenile hormone biosynthesis and signaling

**Fig. 4:**
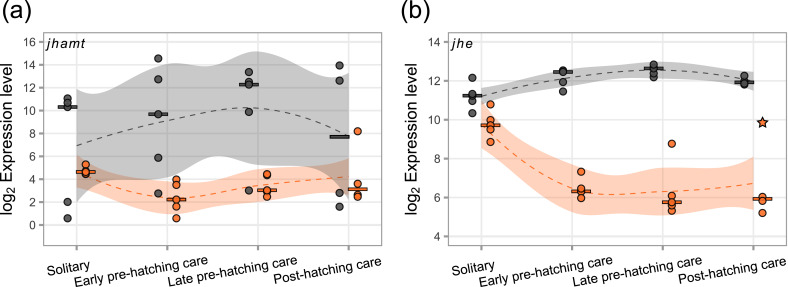
Expression levels of *jhamt* (**a**; *jh acid O-methyltransferase*; LOC108559401) and *jhe (***b**; *jh esterase*; LOC108567338) involved in JH biosynthesis and degradation in the head (grey) and fat body (orange) across a solitary phase and three parental care phases. *jhamt* expression in the head remained stable across phases, while fat body expression changed significantly across phases with the highest expression during the solitary phase (head: LRT-p_*adj*_ = 0.77; fat body: p_*adj*_ < 0.01; star indicates the removed fat body outlier; see methods). In the head, *jhe* expression increased (p_*adj*_ < 0.001), with the highest expression during the late pre-hatching care phase. In the fat body, expression declined with onset of parental care and reached its lowest level during the early pre-hatching care phase (p_*adj*_ < 0.001). Dots represent individual samples and dashed lines (LOESS) show expression pattern with shaded areas indicating the 95% confidence interval. Expression levels are plotted as log_2_ transformed normalized counts. Horizontal bar shows median of samples per phase and tissue

In the Juvenile Hormone biosynthesis pathway, the enzymes JH acid O-methyltransferase (JHAMT) and methyl farnesoate epoxidase (CYP15A1) catalyze the final steps to produce the active juvenile hormone III (JH). Using BLASTp, we identified *N. vespilloides* homologues of the proteins involved in JH biosynthesis in *Tribolium castaneum* (Table S5). Interestingly, we found that genes involved in JH biosynthesis were transcribed in both head and fat body. In the head, the expression levels of the JH synthesizing genes *nves-jhamt* (Fig. 4a) and *nves-cyp15a1* (Fig. S12a) did not significantly change between the parental care phases but tended to increase during pre-hatching care. In the fat body, the expression significantly decreased in the early pre-hatching care phase compared to the solitary phase (*nves-jhamt*: p_*adj*_ < 0.01; *nves-cyp15a1*: p_*adj*_ < 0.001 after removing an outlier from the post-hatching care phase, Fig. 4a, S12).

JH is inactivated through two pathways: either by JH esterase (JHE), which catalyzes the conversion of JH into JH acid, or by JH epoxide hydrolase (JHEH), catalyzing the conversion of JH into JH diol. In the head, *nves-jhe* expression levels increased significantly during the transition from the solitary to the early pre-hatching phase (p_*adj*_ < 0.01; Fig. 4b). In contrast, fat body *nves-jhe* expression levels decreased significantly during the transition from the solitary to the early pre-hatching phase (p_*adj*_ < 0.001), followed by a stable expression pattern throughout pre- and post-hatching care (Fig. 4b). The expression levels of *nves-jheh* were only affected in the fat body. There, expression levels increased significantly during the transition from the solitary to the early pre-hatching phase (p_*adj*_ < 0.01) and tended to decrease slightly in pre- and post-hatching care phases (Fig. S12b).

### Vitellogenesis

**Fig. 5:**
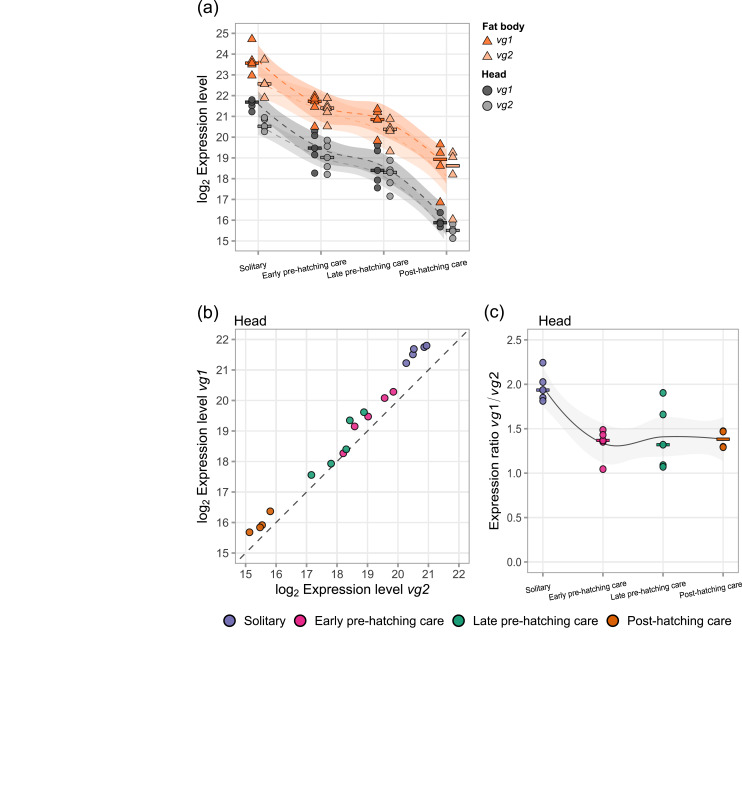
Gene expression levels of *vg1* (*vitellogenin 1*, LOC108564206) and *vg2 (vitellogenin 2*, LOC108565867) across a solitary phase and three parental care phases steadily decreased in both tissues (a; LRT-p_*adj*_ < 0.001). The gene expression of *vg1* and *vg2* was highly correlated in the head (b; negative binomial GLM LRT χ^2^ (1) = 1319.53, *p* < 0.001; the dashed line indicates equal expression levels of *vg1* and *vg2*; note that axes are log-scaled).) and fat body (fig. S9a). In the brood care phases, the gene expression ratio between *vg1* and *vg2* was roughly half of that in the solitary phase in the head (c; GLM LRT F_3,15_ = 8.33, *p* < 0.01), and fat body (fig. S9b). Dots represent individual samples and lines (LOESS) show expression pattern with shaded areas indicating the 95% confidence interval. Expression levels in (**a**) and (**b**) are plotted as log_2_ transformed normalized counts. Horizontal bar shows median of samples per phase and tissue (**a, c**)

Two vitellogenin (*vg*) genes are annotated in the *N. vespilloides* genome [68]. Both *vg1* and *vg2* expression levels were significantly affected by parental care phases in both tissues (Fig. 5a). The expression levels of *vg1* and *vg2* steadily decreased from the solitary phase to the post-hatching care phase, with the biggest change between the solitary and early pre-hatching phase (*vg1*, Head and Fat body: p_*adj*_ < 0.001; *vg2*, Head: p_*adj*_ < 0.001, Fat body: p_*adj*_ < 0.01) and between the late pre-hatching phases and post-hatching care phase (*vg1*, Head: p_*adj*_ < 0.001, Fat body: p_*adj*_ < 0.01; *vg2*, Head: p_*adj*_ < 0.001, Fat body: p_*adj*_ < 0.05) in both tissues. The expression of the two *vitellogenin* copies was highly correlated in both tissues (Head: Fig. 5b, Fat body: Fig. S9a; negative binomial generalized linear model: both tissues *p* < 0.001). Interestingly, the gene expression ratio between *vg1* and *vg2* was significantly lower in the brood care phases compared to the solitary phase in both tissues (Head: Fig. 5c, Fat body: Fig. S9b; generalized linear model: both tissues *p* < 0.01). The expression of the vg receptor gene *vgr* was stable in the head. In the fat body, *vgr* expression was highest in the solitary phase and dipped to a low during early pre-hatching care (p_*adj*_ < 0.001; Fig. S13b), matching the dip in the vg1/vg2 expression ratio in the same tissue (Fig. S9b).

We used a reciprocal BLASTp approach to identify *N. vespilloides* homologues of *T. castaneum* genes involved in the *vitellogenin* regulation (Table S5) [34]). In both tissues we found *protein kinase B* (*nves-akt)* and *forkhead box O* (*nves-foxo) *genes to have a significantly lower expression level in the early pre-hatching care phase compared to the solitary phase (p_*adj*_ < 0.001, Fig. S9c-f, Table S1). We found the expression levels of *vg1* to be correlated with those of *nves-akt* and *nves-foxo* in both tissues (Fig. S9c-f). Expression levels of *nves-akt*, *nves-foxo*, and *vg1* were highest during the solitary phase and decreased thereafter. During the transition to early pre-hatching care, all three genes exhibited a decline in expression across both tissues, which indicated a shared regulatory pattern at this stage. In subsequent phases, *vg1* expression continued to decrease, whereas *nves-akt* and *nves-foxo* expression levels stabilized and no longer showed significant changes, resulting in a decoupling of their expression patterns (Fig. S9c-f).

## Discussion

We analyzed the gene expression of *Nicrophorus vespilloides* females throughout the reproductive cycle and found that the strongest shift in gene expression occurred when the beetles moved onto the breeding resource. Metabolism-related gene expression increased, whereas that related to translation and protein biosynthesis declined. At the same time, JH and IIS signaling decreased, leading to reduced *vitellogenin* expression relative to the high levels present prior to egg laying. This pattern indicates that beetles switch from investment into the future (accumulating energy reserves, preparing egg laying) to expending resources for the current and likely final reproduction event by providing for their offspring. Compared to the major transition in gene expression at the onset of care, we found relatively few differences between the three parental care phases, suggesting a persistent molecular state with only small regulatory adjustments. These adjustments mostly take place once beetles interact with their larvae, and most prominently feature genes presumably involved in social immunity.

### Increased metabolism upon carcass discovery

Our study reveals that the discovery and burial of a carcass is associated with the upregulation of genes involved in metabolic, mitochondrial, and peroxisomal activities. We suggest that this upregulation is primarily driven by (i) increased feeding [57] and digestion associated with resource acquisition, and by (ii) a large demand for energy caused by early parental care behaviors such as burying. When females bury a carcass, they initially feed and gain weight [57]. During later parental care phases, the metabolism genes were downregulated and indeed it is known that at this time the parental beetles lose weight even though food remains abundant [57]. This is likely because they regurgitate a substantial portion of their intake to feed their offspring, while at the same time facing high metabolic demands. In breeding *Nicrophorus orbicollis*, this is reflected in an increased CO2 production compared to non-breeding controls [28]. The expression of genes associated with translational activity was downregulated once beetles began to prepare the carcass. This is additional evidence that instead of synthesizing proteins that help with body maintenance, beetles directly invest most of the consumed resources into brood care [69]. Individuals have to trade off investment between body maintenance and reproduction. Because carcasses are scarce, each breeding event likely represents the ultimate investment for female *N. vespilloides*. Consequently, individuals may be selected to increase their current parental investment, at the expense of physiological functions that would pay off only later. Reduced translational activity may therefore indicate a reduced investment into body maintenance. Similarly, reduced *vg* expression during brood care may indicate that females do not invest in oogenesis for potential future reproduction while they are already caring for a batch of offspring. That the trade-off is tipped towards current reproduction is supported by data showing that, after oviposition, ovarian weight is reduced [70], oviposition is suppressed during the offspring provisioning, and that breeding females have shorter lifespans than non-breeding female burying beetles [23, 71].

### Vitellogenin and the regulation of reproduction

Our data showed that *vg* expression decreases significantly in both the head and fat body across the parental care phases and is highest in mated, non-breeding females (Fig. 5a). This supports the idea that vitellogenin is produced and stored in the hemolymph before a reproduction resource is even detected, allowing females to lay eggs rapidly once a carcass becomes available [39]. This pre-reproductive priming likely evolved in response to the ephemeral nature of and intense competition for carcasses [52]. By synthesizing and storing vitellogenin in advance, females can initiate egg production immediately and shift rapidly to carcass preparation, reducing the risk of losing the resource to quicker competitors. After egg laying, Vg is no longer required, explaining its decline in expression once females begin parental care.

Interestingly, we found *vg* expression in the head samples to be strongly correlated with its expression in the fat body, suggesting that the abdominal fat body is not the only location of Vg production. Furthermore, we found that *nves-akt* and *nves-foxo* expression were strongly correlated with *vg1* expression (Fig. S9c-f), as would be predicted if an activation of the IIS pathway induced Vg production [34]. As IIS is a nutrient-sensing pathway, beetles may produce vitellogenin whenever they take in enough food on larger carcasses where they do not reproduce. In our study, IIS activation and *vg* transcription dropped when beetles prepared for brood care. Interestingly, the ratio between *vg1* and *vg2* expression dropped when beetles entered brood care phase (Fig. 5c), which could potentially indicate incipient regulatory specialization of the two copies. In social Hymenoptera, *vitellogenin* genes have duplicated multiple times, and different copies regulate different behavioral functions, including brood care [72, 73].

### Social immunity

We found that the expression of several genes encoding antimicrobial peptides, lysozymes, and peptidoglycan recognition proteins increased sharply when the beetles anticipated the hatching of larvae (Fig. 3). The beetles produce proteins and enzymes that help them to preserve carcass quality and to reduce the load of harmful microbes, and thereby provide social immunity which has been shown to be beneficial for burying beetle offspring development [53, 54, 74].

In addition, we found an interesting peak in *nves-thau4* and *nves-clys2* expression, and generally of immune pathways, during post-hatching care (Fig. 3a, b). The corresponding peptides are known to be excreted through anal secretions only when beetles are on a carcass [55]. Our finding that their expression is further upregulated once the beetles begin direct brood care suggests a social role of these immune effectors. The peak in immune pathway gene expression during post-hatching care (Fig. 3) raises the question whether some immune related effectors are transferred to the larvae by feeding. In *N. orbicollis,* larvae cannot survive without post-hatching care and offspring survival rate increases if fed with parental oral fluids, suggesting that beneficial compounds are transferred to the offspring [74, 75]. Oral transfer of immune effectors, hormones, and other regulatory elements has been identified in many species [76–78]. An alternative explanation for the late peak in the expression of some immune genes might be that microbial challenges increase as the carcass ages. To properly disentangle potential social immunity effects from those caused by the progressively ageing carcass would require targeted experiments.

### Takeout and neuropeptide F

In our transcriptomic analyses, we investigated candidate regulators of care behavior in burying beetles that had been highlighted in previous transcriptomic studies. The expression patterns we observed generally support earlier findings, while also providing further insight on their potential roles in regulating care. In burying beetles, both Takeout and NPF emerged as candidate regulators of parental behavior [46, 49]. We found the *N. vespilloides takeout* homologue of *Drosophila melanogaster* to display similar expression patterns to those reported in *N. orbicollis*, where they might mediate temporal kin recognition [49]. Head expression levels peaked during the late pre-hatching care phase, which coincides with larval acceptance (Fig. S11). Neuropeptide F increases feeding motivation in solitary insects [79]. In insects that feed their offspring, it appears to have been shifted from regulating self-feeding to mediating offspring provisioning. Our results are in accord with this idea as *nves-npfr* expression decreased with intensification of parental care, which would redirect the motivation away from self-feeding and toward offspring provisioning (Fig. S10b).

### JH biosynthesis and regulation

Juvenile hormone titers peak twice during burying beetle parental care, once upon detection of the carcass and once when the beetles begin to feed their larvae [22, 23]. Given that JH is synthesized in the *corpora allata* (CA) and rapidly distributed throughout the body via the hemolymph, we expected the expression of JH biosynthesis-related genes *nves-jhamt* and *nves-cyp15a1* in the head to reflect the hormone titers previously measured across parental care stages. This was not the case. In particular the second JH peak during the post-hatching care phase was not evident in the expression of JH biosynthesis genes (Fig. 4a, S12a). This might indicate that gene expression changes in JH biosynthesis are transient and were missed in our sampling, or that the production and the release of JH are decoupled.

A second important factor in the regulation of JH titers is the breakdown of the JH molecule by enzymes like JHE and JHEH. Head expression levels of *nves-jhe* significantly increased in the early and late pre-hatching care phase compared to the solitary phase, indicating a quicker degradation of JH during parental care, and thus potentially lower JH titers (Fig. 4b). However, the literature indicates that the fat body is the main source of JHE secreted into the hemolymph [80, 81]. Thus, the downregulation of JHE expression we observed in the fat body during post-hatching care could explain the high JH titers reported for this phase (Fig. S4b [23]).

JH typically affects the expression of *krüppel homolog (kr-h1*). However, *nves-kr-h1* expression remained stable regardless of the parental care phases (Fig. S13a). The CA synthesizes JH on demand, and it diffuses rapidly into the hemolymph after production. JH signaling may thus be relatively stable and only interrupted by transcriptional pulses induced by key events such as carcass discovery and the transition to direct interactions with larvae. Our sampling might have missed these pulses.

### Potential communicative function of the JH pathway

Interestingly, the two JH biosynthesis genes *nves-jhamt* and *nves-cyp15a1* were not only expressed in the head but also in the fat body. This suggests that JH production may not be exclusive to the CA, as some reports already indicated [82–84]. An alternative explanation is that in the fat body, the JH biosynthesis pathway may have been co-opted for the production of other substances. In burying beetles, the volatile compound (E)-methyl-geranate (MG) is emitted predominantly by breeding females and serves as a chemical signal reflecting their hormonal state during parental care [85]. The emission of MG is positively correlated with JH titers, indicating its role as a reliable indicator of hormonal status. The release of MG by females during intensive parental care, when they are temporarily infertile, deters male mating and functions as an anti-aphrodisiac [23]. Among burying beetles, MG is evolutionary conserved suggesting that the pheromone plays an important role in social evolution [86]. MG and JH are terpenoids, which are typically synthesized through the mevalonate pathway that includes JHAMT [23]. It has been hypothesized that MG is a by-product of the JH biosynthesis and was co-opted for signaling during the evolution of biparental care in burying beetles [23, 86]. It is thus tempting to speculate that the genes coding for enzymes involved in JH synthesis are expressed to produce enzymes involved in the production of MG.

## Conclusions

The transition from solitary life to parental care is accompanied by dramatic changes in gene expression, while the differences between the distinct care phases are comparatively subtle. We propose that this pattern reflects an adaptation to the ecological pressures of a highly contested and short-lived resource. Burying beetles must act quickly to secure and exploit the carcass, which may explain the immediate and extensive transcriptional regulation of metabolism and immunity observed at the onset of breeding. In addition, these early molecular changes likely serve to prepare individuals for the demands of post-hatching care. Because the carcass is such an ephemeral resource, the eggs develop rapidly, leaving the parents with little time to prepare for feeding their offspring [61, 62]. Our findings indicate that parental care in *N. vespilloides* is orchestrated by a coordinated set of molecular and hormonal pathways, with several potential regulators such as vitellogenins and neuropeptides contributing to the transition between solitary life and brood care.

## Electronic supplementary material

Below is the link to the electronic supplementary material.

Supplementary material 1

Supplementary material 2

Supplementary material 3

Supplementary material 4

Supplementary material 5

Supplementary material 6

## Data Availability

Raw sequence data are available from the European Nucleotide Archive under the accession number PRJEB104015.
