# Supplementary material for: Preparing for parenthood: molecular reprogramming precedes parenting in the burying beetle Nicrophorus vespilloides
Source: Front Zool. 2026 May 18;23:22. doi: 10.1186/s12983-026-00615-4 (PMC13188450; doi:10.1186/s12983-026-00615-4)
Supplement: Supplementary file 1 — Supplementary material 1 [file 12983_2026_615_MOESM1_ESM.pdf]

Preparing for parenthood: molecular reprogramming precedes parenting in the burying beetle *Nicrophorus vespilloides*

Supplemental Methods

Tissue Collection

After we collected the females of each phase, we immediately separated whole heads, including antennae, from the thorax using scissors and transferred them into a mortar containing liquid nitrogen. The tissue was then grounded with a pestle, and the resulting powder was collected and transferred into 600 µl of TRI Reagent®. For fat body collection, a petri dish ("Blockschälchen") was placed on ice, and the beetle’s abdomen was opened along the epipleuron and immersed in ice-cold 1× PBS. Using a binocular microscope, the gut, ovaries, and trachea were removed and discarded. The anterior fat body was then carefully dissected with tweezers and transferred into 600 µl of TRI Reagent®. Head and abdomen tissues were always dissected from the same individual beetles. Both tissue types were separately homogenized using a high-speed tissue homogenizer (TissueLyser II, Qiagen) for 2-3 minutes at 25 Hz with 2.0 mm zirconia beads. The homogenates were then incubated for 10 minutes on a shaker at room temperature. To remove particulate debris, the homogenates were vortexed, centrifuged, and the supernatant was transferred into a new nuclease-free tube. RNA isolation was performed according to the manufacturer’s instructions using the Direct-zol™ RNA MiniPrep Kit (Zymo). Finally, RNA was eluted in 40 µl of RNase/DNase-free water. RNA concentration and purity were measured using a NanoDrop™ 2000c Spectrophotometer (Thermo Scientific™). RNA samples were stored at –80°C until shipment for sequencing. The samples were sent on dry ice to Beijing Genomics Institute (BGI) Tech Solutions Co. (Hong Kong). Overall, we collected five head and five fat body samples for each phase, except during the post-hatching care phase, where we obtained only four samples per tissue because of bad RNA quality in one sample.

Sequencing

RNA concentration and quality were assessed at BGI using an Agilent 2100 Bioanalyzer before library preparation and sequencing. Transcriptomic data were

obtained through paired-end 150 bp sequencing on an Illumina HiSeq X Ten platform, yielding approximately 4.95 Gb per sample.

Quality Control and Data Processing

Raw sequencing data quality was assessed using FastQC v.0.12.1 (Andrews, 2010) and summarized with MultiQC v.1.17 (Ewels et al., 2016). Adapter sequences were trimmed using fastp v.0.23.4 (Chen et al., 2018). The cleaned reads were then mapped to the *Nicrophorus. vespilloides* genome (RefSeq: GCF_001412225.1_Nicve_v1.0) obtained from the National Center for Biotechnology Information (NCBI), using HISAT2 v.2.2.1 (Kim et al., 2019) with a mean overall alignment rate of 85 % (Head: 83.4 %; Fat body: 86.6 %). SAM files generated from the mapping were converted to read-name sorted BAM files with SAMtools v.1.20 (Danecek et al., 2021). Read counts per gene for each sample were then quantified with the htseq-count function in HTSeq v.2.0.5 (Anders et al., 2015) and merged into a single dataset for downstream analysis.

Downstream data analysis

Data was processed, analyzed, and plotted using R Statistical Software 4.4.1 (R Core Team, 2025) within the RStudio environment (Posit team, 2024) with the loaded packages: Tidyverse v2.0.0 (Wickham et al., 2019), ggrepel v0.9.6 (Slowikowski, 2024), MASS v7.3-60.2 (Venables & Ripley, 2002), colorspace v2.1-1 (Zeileis et al., 2009).

Principal Component Analysis

We performed a principal component analysis (PCA) to assess global differences between samples using normalized gene expression data. The analysis was conducted on regularized log transformed counts generated with the *rlog* function, followed by a PCA using plotPCA from DESeq2 v.1.44.0 (Love et al., 2014).

Functional Enrichment Analysis and Gene annotation

To summarize the functions of the differentially expressed genes, we conducted a functional pathway enrichment analysis using Kyoto Encyclopedia of Genes and Genomes (KEGG) terms on pairwise comparisons between successive phases. We used the enrichKEGG function in clusterProfiler v.4.12.6 (Xu et al., 2024), specifying the “ncbi-geneid” keyType and KEGG organism code “nvl.” KEGG terms with an adjusted p-value < 0.05, calculated via the Benjamini & Hochberg method, were

considered significantly enriched (Tab. S3). We obtained functional annotations of genes using InterProScan v.5.63-95.0 (Jones et al., 2014), NCBI-RefSeq, and UniProt/Swiss-Prot reference proteomes with the *N. vespilloides* proteome from NCBI (GCF_001412225.1_Nicve_v1.0) as input. In addition, we used the gene annotations based on the *N. vespilloides* genome on NCBI. We used DIAMOND BLASTp (Buchfink et al., 2021) with an e-value threshold of 0.001. The top BLAST hit is reported in Table S1 and S2. If the top hit lacked a meaningful annotation (e.g. "uncharacterized protein"), we repeated the BLASTp search with a relaxed e-value threshold of 0.01 and the *ultra-sensitive* option. The top hit with a meaningful description was then selected and is reported in Table S1 and S2. BLASTp was used to identify antimicrobial peptide (AMP) and lysozyme sequences in *N. vespilloides* reported by (Jacobs et al., 2016) and peptidoglycan recognition proteins (PGRPs) based on the functional annotations. We extracted Protein family (Pfam) domain information was extracted from InterProScan output to infer protein function (Tab. S1 and S2). We conducted reciprocal BLASTp searches to investigate the presence of proteins in *N. vespilloides* that are involved in vitellogenin expression and regulation, JH biosynthesis, and signaling pathways in *Tribolium castaneum*, as well as homologues of the Takeout protein from *Drosophila melanogaster*. Hits with ≥ 30 % similarity and an e-value < 0.001 were retained, and the highest-scoring reciprocal match was designated as the putative homologue (Tab. S5).

References

Anders, S., Pyl, P. T., & Huber, W. (2015). HTSeq—A Python framework to work with high-throughput sequencing data. *Bioinformatics*, *31*(2), 166–169. https://doi.org/10.1093/bioinformatics/btu638

Andrews, S. (2010). *FastQC: a quality control tool for high throughput sequence data.* http://www.bioinformatics.babraham.ac.uk/projects/fastqc

Buchfink, B., Reuter, K., & Drost, H.-G. (2021). Sensitive protein alignments at tree-of-life scale using DIAMOND. *Nature Methods*, *18*(4), 366–368. https://doi.org/10.1038/s41592-021-01101-x

Chen, S., Zhou, Y., Chen, Y., & Gu, J. (2018). fastp: An ultra-fast all-in-one FASTQ preprocessor. *Bioinformatics*, *34*(17), i884–i890. https://doi.org/10.1093/bioinformatics/bty560

Danecek, P., Bonfield, J. K., Liddle, J., Marshall, J., Ohan, V., Pollard, M. O., Whitwham, A., Keane, T., McCarthy, S. A., Davies, R. M., & Li, H. (2021). Twelve years of SAMtools and BCFtools. *GigaScience*, *10*(2). https://doi.org/10.1093/gigascience/giab008

Ewels, P., Magnusson, M., Lundin, S., & Käller, M. (2016). MultiQC: summarize analysis results for multiple tools and samples in a single report. *Bioinformatics*, *32*(19), 3047–3048. https://doi.org/10.1093/bioinformatics/btw354

Jacobs, C. G. C., Steiger, S., Heckel, D. G., Wielsch, N., Vilcinskas, A., & Vogel, H. (2016). Sex, offspring and carcass determine antimicrobial peptide expression in the burying beetle. *Scientific Reports*, *6*(1), 25409. https://doi.org/10.1038/srep25409

Jones, P., Binns, D., Chang, H. Y., Fraser, M., Li, W., McAnulla, C., McWilliam, H., Maslen, J., Mitchell, A., Nuka, G., Pesseat, S., Quinn, A. F., Sangrador-Vegas, A., Scheremetjew, M., Yong, S. Y., Lopez, R., & Hunter, S. (2014). InterProScan 5: Genome-scale protein function classification. *Bioinformatics*, *30*(9), 1236–1240. https://doi.org/10.1093/BIOINFORMATICS/BTU031

Kim, D., Paggi, J. M., Park, C., Bennett, C., & Salzberg, S. L. (2019). Graph-based genome alignment and genotyping with HISAT2 and HISAT-genotype. *Nature Biotechnology*, *37*(8), 907–915. https://doi.org/10.1038/s41587-019-0201-4

Love, M. I., Huber, W., & Anders, S. (2014). Moderated estimation of fold change and dispersion for RNA-seq data with DESeq2. *Genome Biology*, *15*(12), 550. https://doi.org/10.1186/s13059-014-0550-8

Posit team. (2024). *RStudio: Integrated Development Environment for R*. Posit Software, PBC, Boston, MA. http://www.posit.co/

R Core Team. (2025). *R: A Language and Environment for Statistical Computing*. R Foundation for Statistical Computing, Vienna, Austria. https://www.r-project.org/

Slowikowski, K. (2024). *ggrepel: Automatically Position Non-Overlapping Text Labels with "ggplot2".* https://CRAN.R-project.org/package=ggrepel

Venables, W. N., & Ripley, B. D. (2002). *Modern Applied Statistics with S*. Springer New York. https://doi.org/10.1007/978-0-387-21706-2

Wickham, H., Averick, M., Bryan, J., Chang, W., McGowan, L., François, R., Grolemund, G., Hayes, A., Henry, L., Hester, J., Kuhn, M., Pedersen, T., Miller, E., Bache, S., Müller, K., Ooms, J., Robinson, D., Seidel, D., Spinu, V., … Yutani, H. (2019). Welcome to the Tidyverse. *Journal of Open Source Software*, *4*(43), 1686. https://doi.org/10.21105/joss.01686

Xu, S., Hu, E., Cai, Y., Xie, Z., Luo, X., Zhan, L., Tang, W., Wang, Q., Liu, B., Wang, R., Xie, W., Wu, T., Xie, L., & Yu, G. (2024). Using clusterProfiler to characterize multiomics data. *Nature Protocols*, *19*(11), 3292–3320. https://doi.org/10.1038/s41596-024-01020-z

Zeileis, A., Hornik, K., & Murrell, P. (2009). Escaping RGBland: Selecting colors for statistical graphics. *Computational Statistics & Data Analysis*, *53*(9), 3259–3270. https://doi.org/10.1016/j.csda.2008.11.033

Supplemental Figures

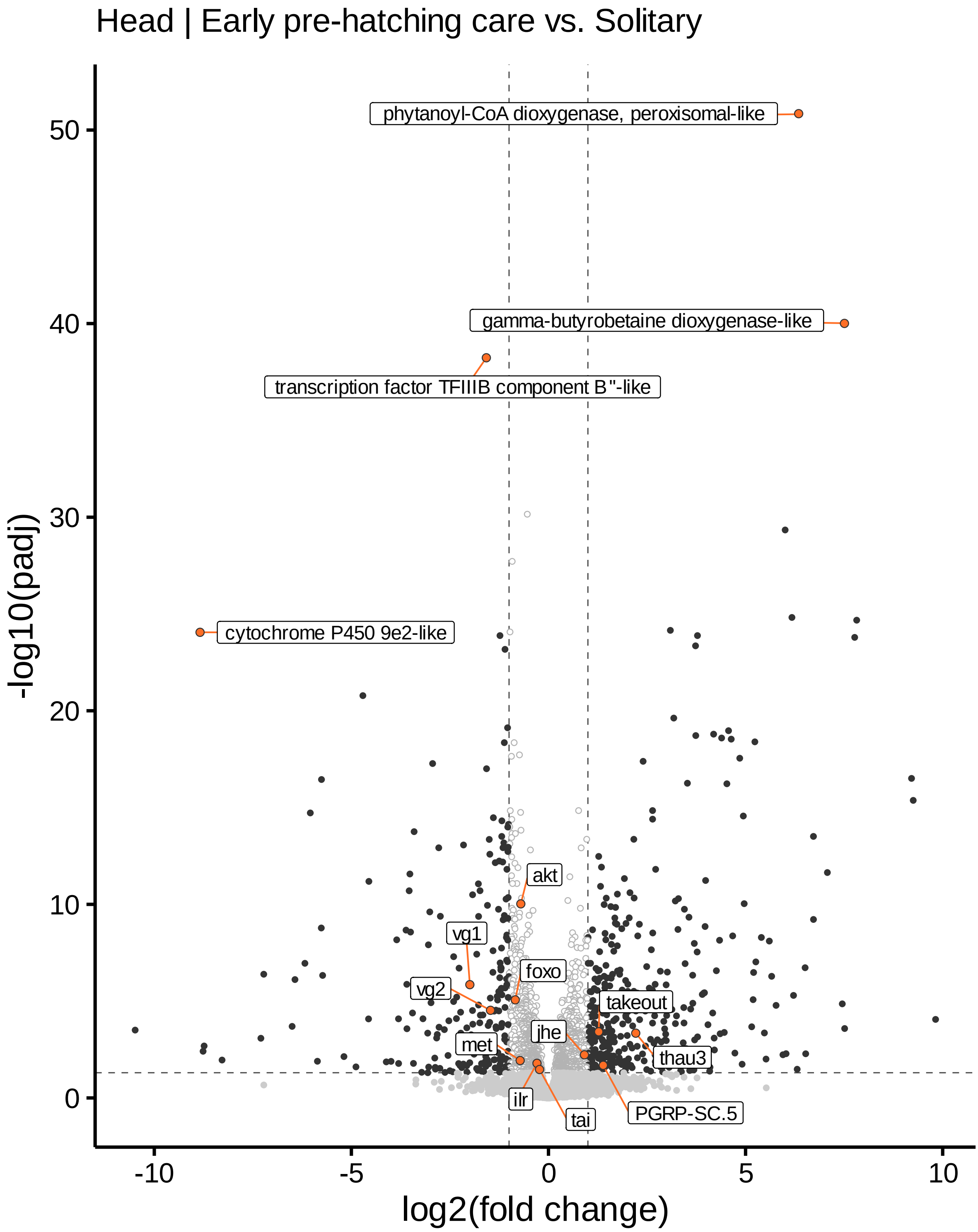

Figure S1: Gene expression changes between the early pre-hatching care and the solitary phase in the head. Each dot represents a gene expressed in the head. Orange dots, labeled with gene names, highlight genes of particular interest or those showing especially strong regulation. Black dots indicate significantly regulated genes (log_2(FC) < -1 or > 1; p_{adj} < 0.05). White dots with grey outlines represent genes with significant but weaker regulation (log_2(FC) > -1 or < 1; p_{adj} < 0.05). Grey dots represent genes without significant regulation (p_{adj} ≥ 0.05).

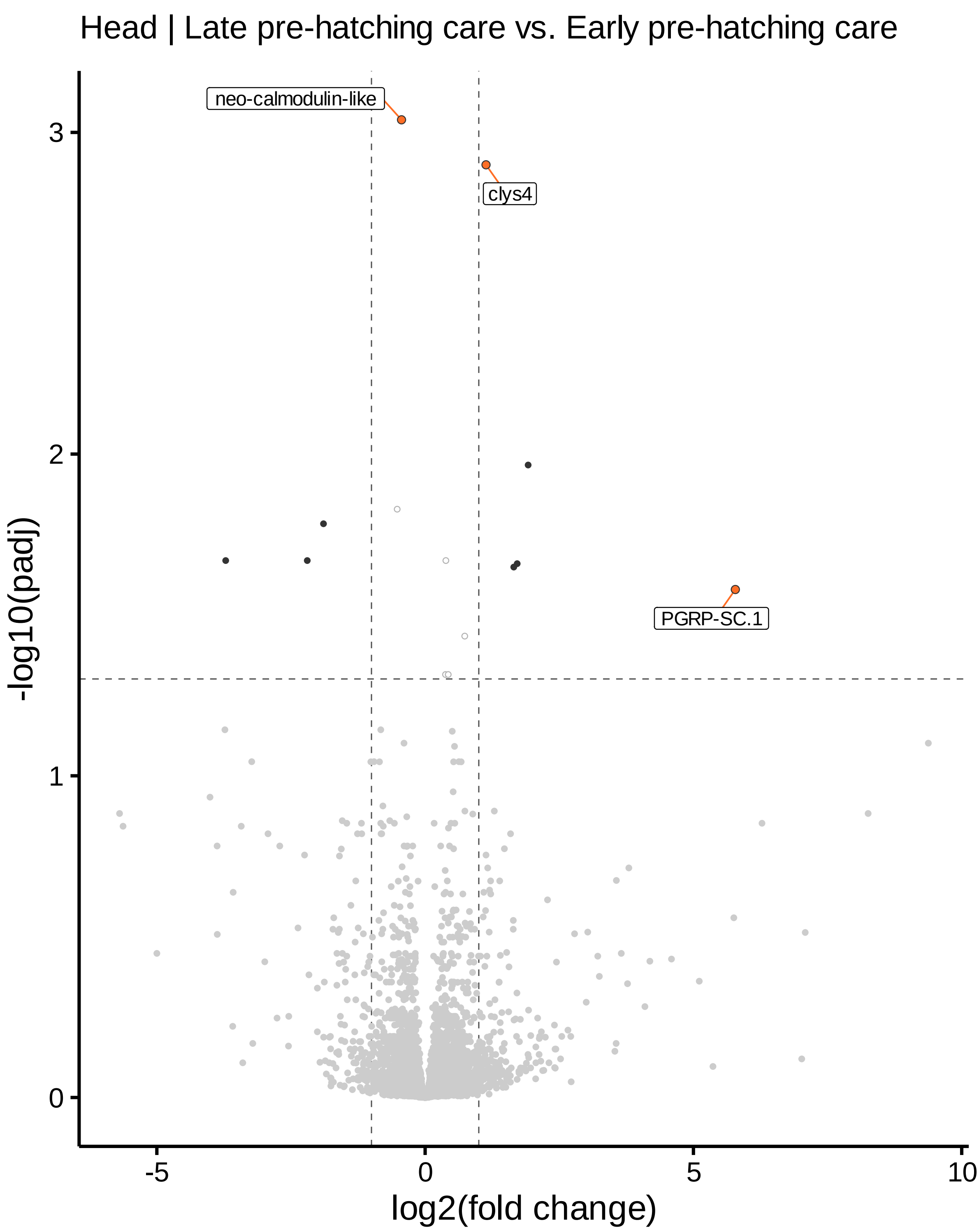

Figure S2: Gene expression changes between the late and the early pre-hatching care phases in the head. Each dot represents a gene expressed in the head. Orange dots, labeled with gene names, highlight genes of particular interest or those showing especially strong regulation. Black dots indicate significantly regulated genes ($log_2(FC) < -1$ or > 1; $p_{adj} < 0.05$). White dots with grey outlines represent genes with significant but weaker regulation ($log_2(FC) > -1$ or < 1; $p_{adj} < 0.05$). Grey dots represent genes without significant regulation ($p_{adj} \geq 0.05$).

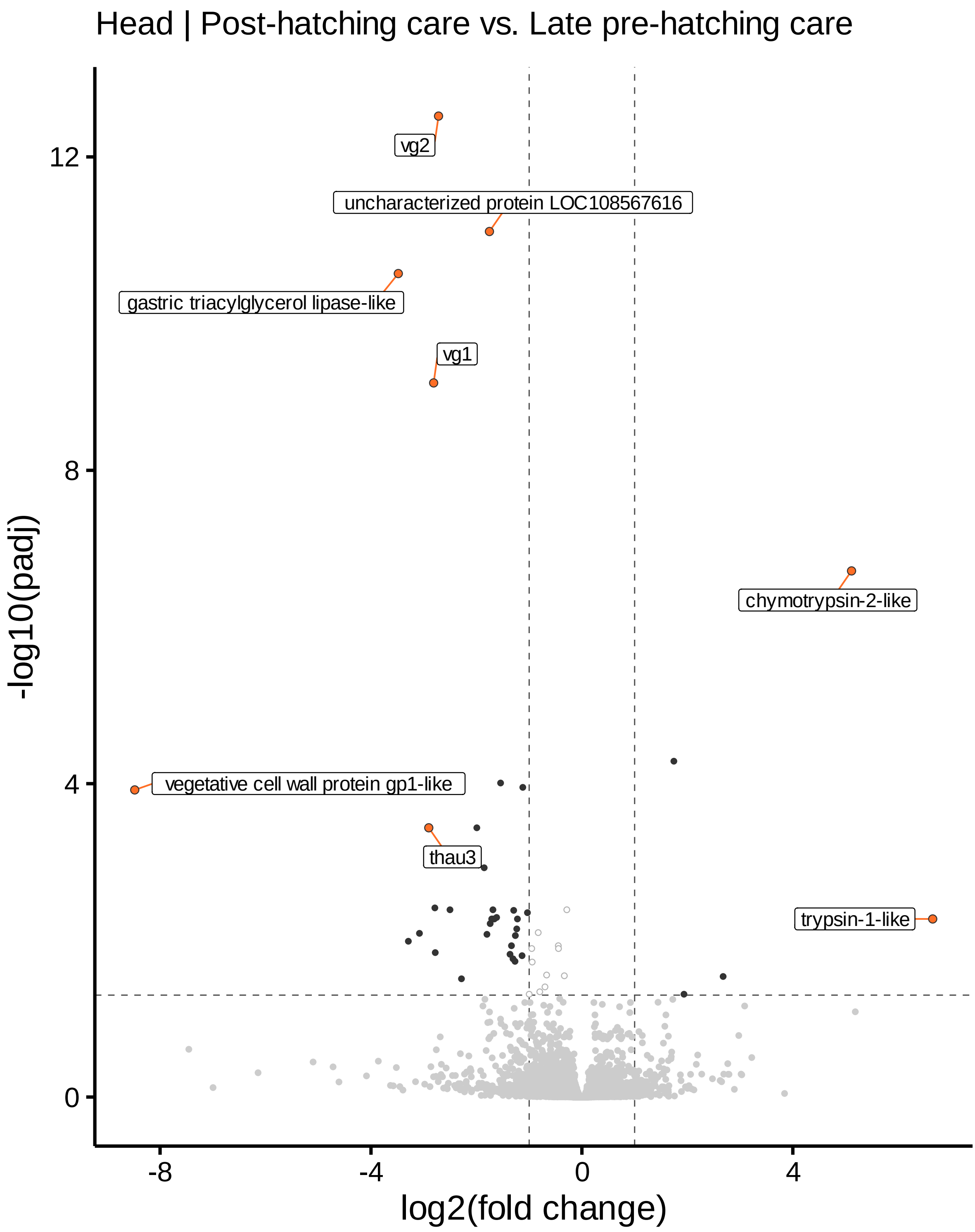

Figure S3: Gene expression changes between the post-hatching and the late pre-hatching care phase in the head. Each dot represents a gene expressed in the head. Orange dots, labeled with gene names, highlight genes of particular interest or those showing especially strong regulation. Black dots indicate significantly regulated genes ($log_2(FC) < -1$ or > 1; $p_{adj} < 0.05$). White dots with grey outlines represent genes with significant but weaker regulation ($log_2(FC) > -1$ or < 1; $p_{adj} < 0.05$). Grey dots represent genes without significant regulation ($p_{adj} \geq 0.05$).

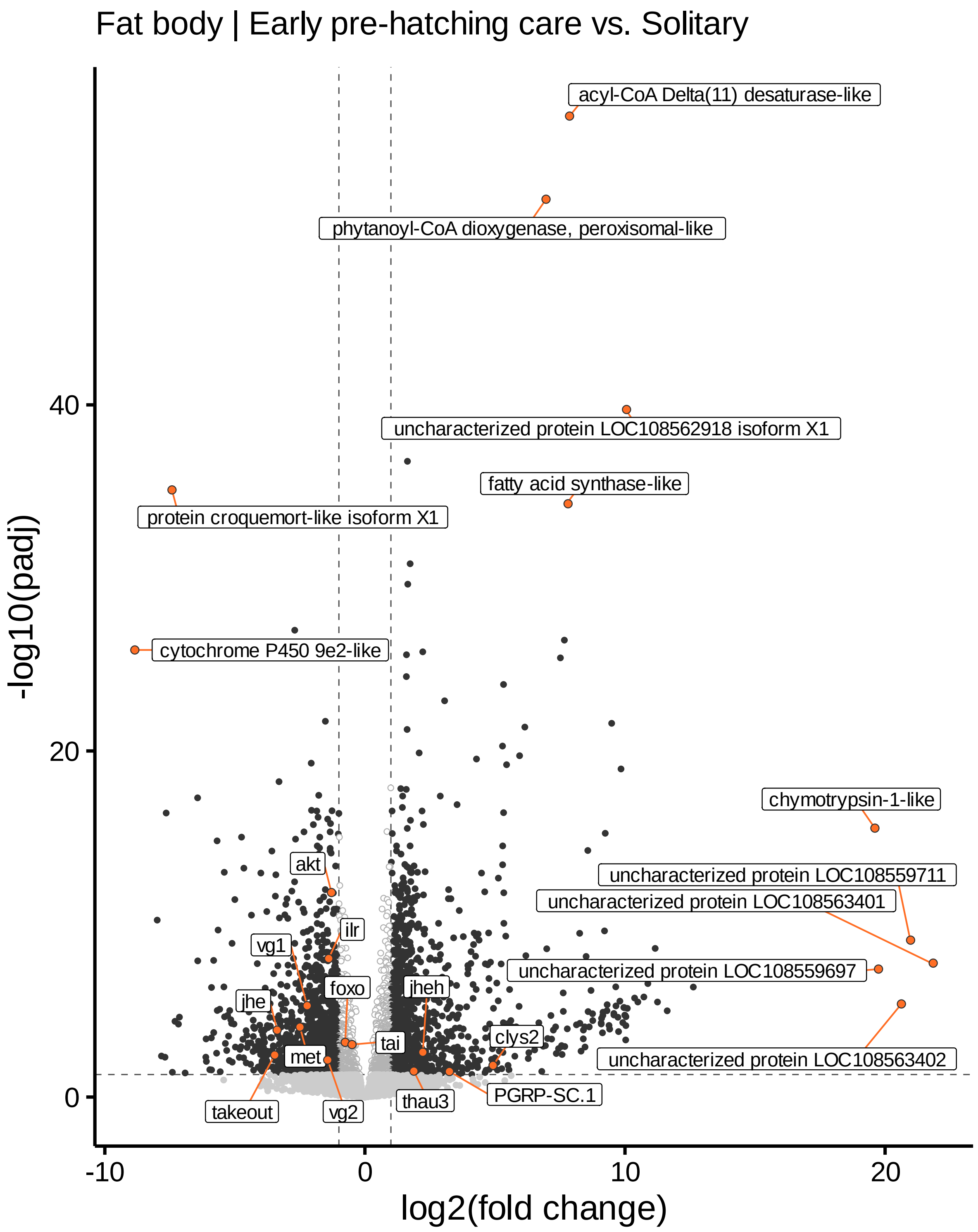

Figure S4: Gene expression changes between the early pre-hatching care and the solitary phase in the fat body. Each dot represents a gene expressed in the fat body. Orange dots, labeled with gene names, highlight genes of particular interest or those showing especially strong regulation. Black dots indicate significantly regulated genes (log_2(FC) < -1 or > 1; p_{adj} < 0.05). White dots with grey outlines represent genes with significant but weaker regulation (log_2(FC) > -1 or < 1; p_{adj} < 0.05). Grey dots represent genes without significant regulation ($p_{adj} \geq 0.05$).

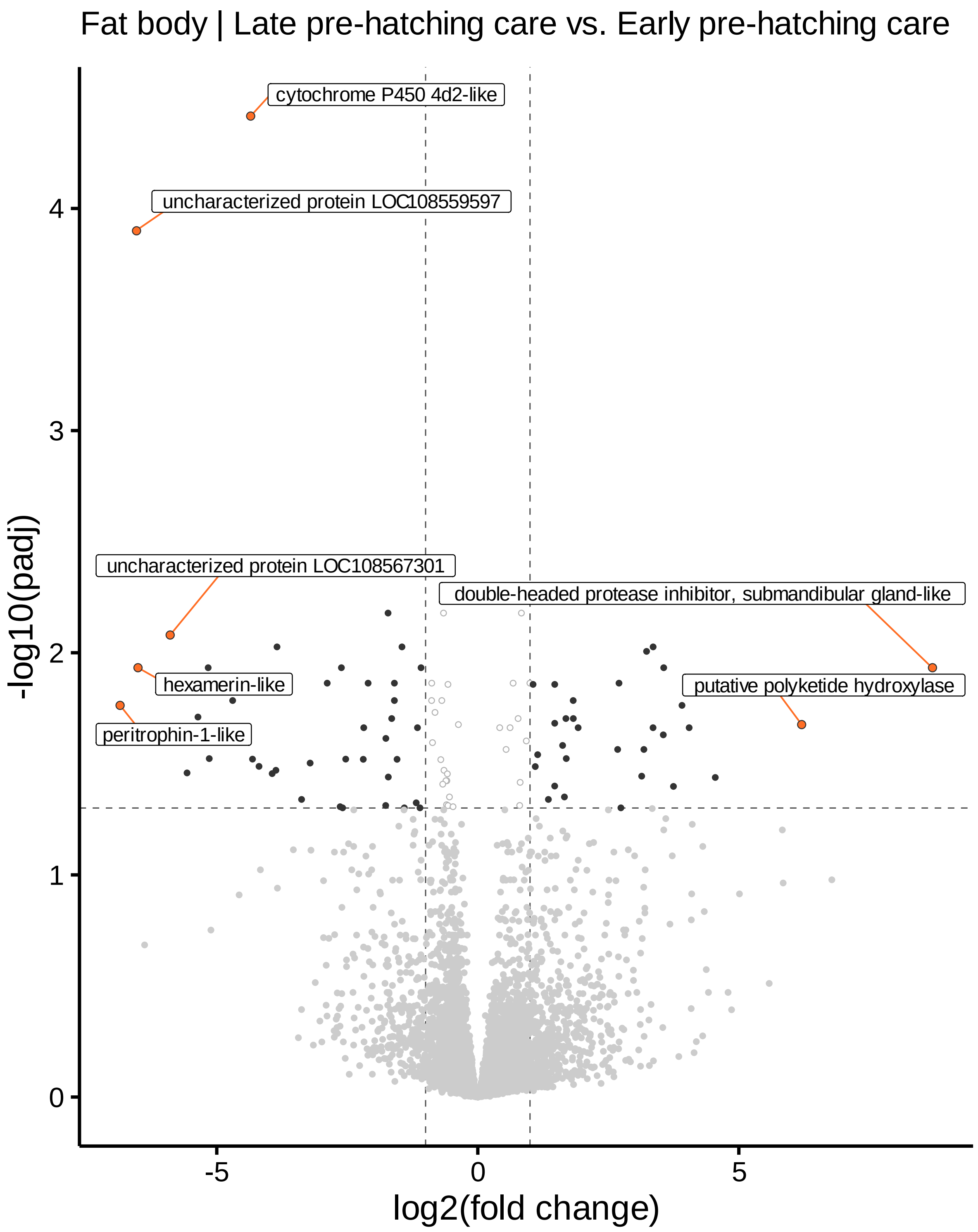

Figure S5: Gene expression changes between the late and the early pre-hatching care phase in the fat body. Each dot represents a gene expressed in the fat body. Orange dots, labeled with gene names, highlight genes of particular interest or those showing especially strong regulation. Black dots indicate significantly regulated genes (log_2(FC) < -1 or > 1; p_{adj} < 0.05). White dots with grey outlines represent genes with significant but weaker regulation (log_2(FC) > -1 or < 1; p_{adj} < 0.05). Grey dots represent genes without significant regulation ($p_{adj} \geq 0.05$).

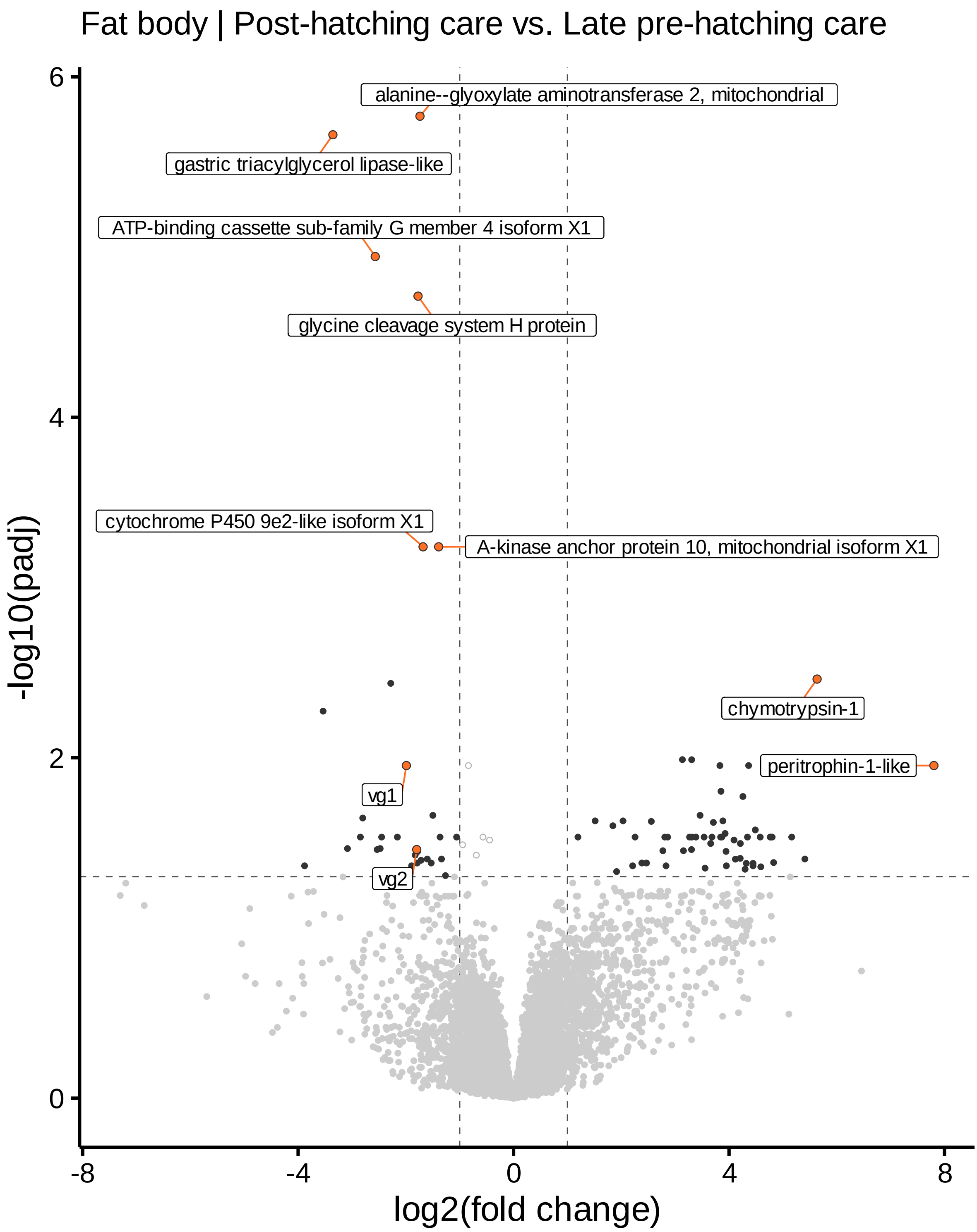

Figure S6: Gene expression changes between the post-hatching and the late pre-hatching care phase in the fat body. Each dot represents a gene expressed in the fat body. Orange dots, labeled with gene names, highlight genes of particular interest or those showing especially strong regulation. Black dots indicate significantly regulated genes (log_2(FC) < -1 or > 1; p_{adj} < 0.05). White dots with grey outlines represent genes with significant but weaker regulation (log_2(FC) > -1 or < 1; p_{adj} < 0.05). Grey dots represent genes without significant regulation (p_{adj} ≥ 0.05).

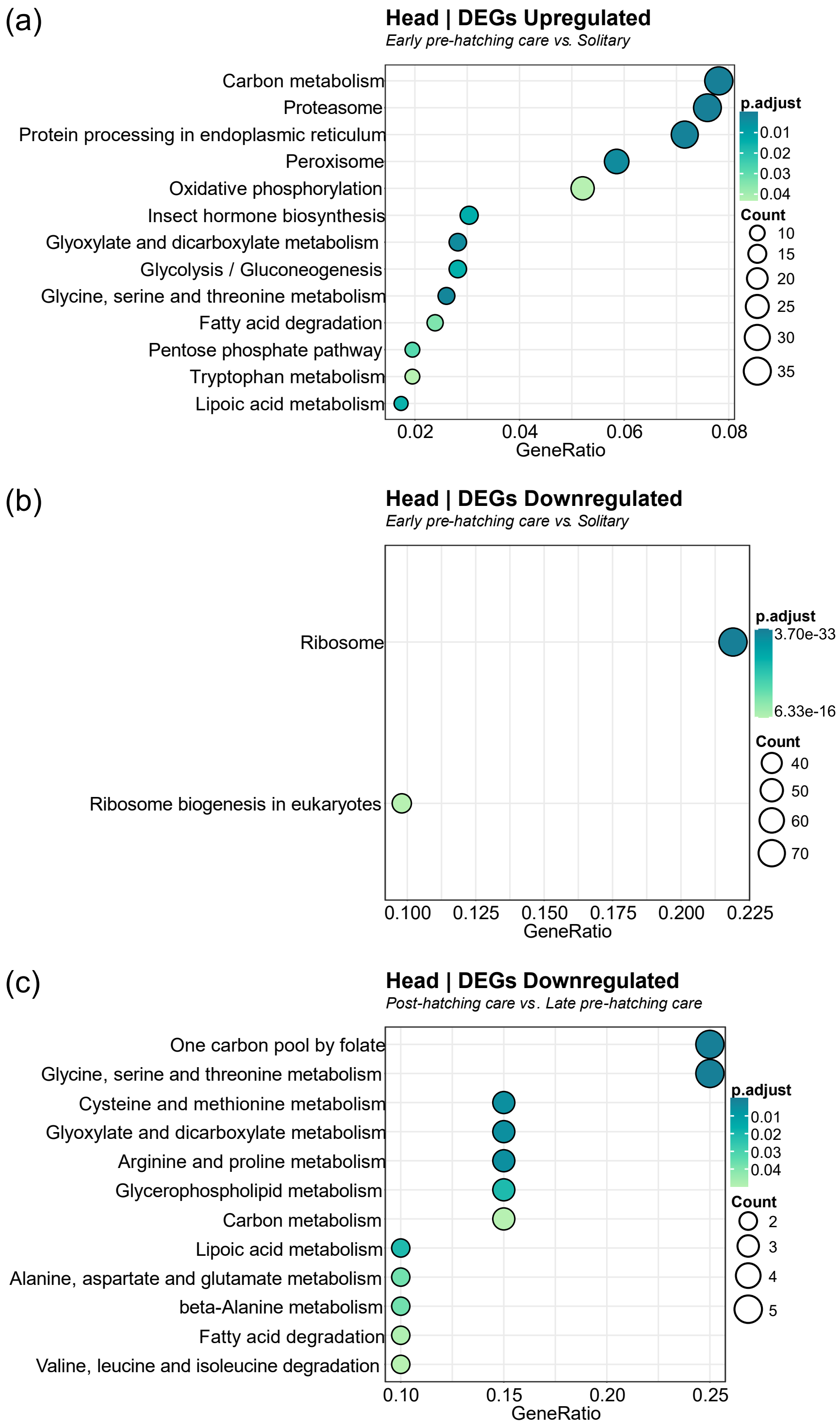

Figure S7: KEGG pathway enrichment analysis for the head tissue. Significantly enriched KEGG pathways were identified separately among up- and downregulated DEGs for pairwise phase comparisons (a - c). Each dot corresponds to a particular pathway, where the x-axis displays the Gene Ratio (ratio of genes annotated to the term relative to the total number of genes). The size of each dot reflects the number of genes associated with that pathway. The color gradient of the dots represents the adjusted p-value for statistical significance of enrichment. Only the pairwise comparisons of subsequent phase transitions that yielded significant enrichment are shown.

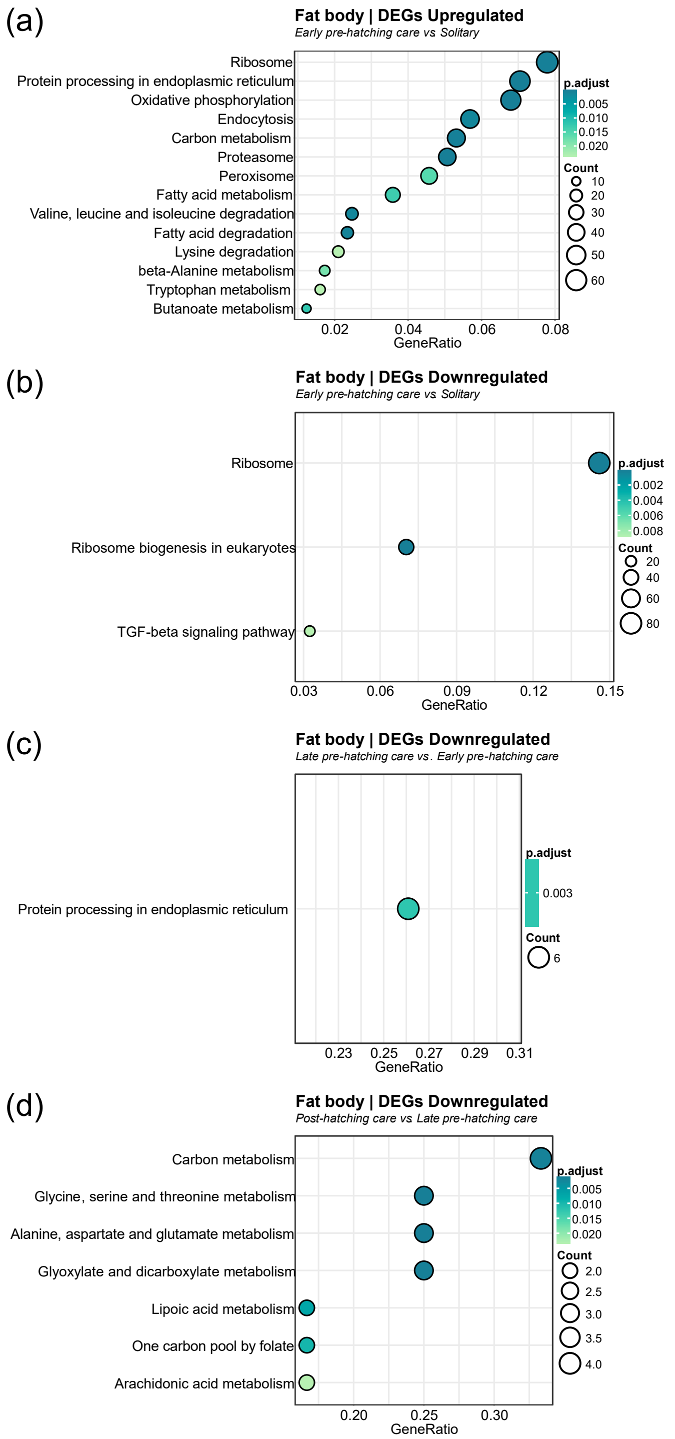

Figure S8: KEGG pathway enrichment analysis for the fat body tissue. Significantly enriched KEGG pathways were identified separately among up- and downregulated DEGs for pairwise phase comparisons in the fat body (a - d). Each dot corresponds to a particular pathway, where the x-axis displays the Gene Ratio (ratio of genes annotated to the term relative to the total number of genes). The size of each dot reflects the number of genes associated with that pathway. The color gradient of the dots represents the adjusted p-value for statistical significance of enrichment. Only the pairwise comparisons of subsequent phase transitions that yielded significant enrichment are shown.

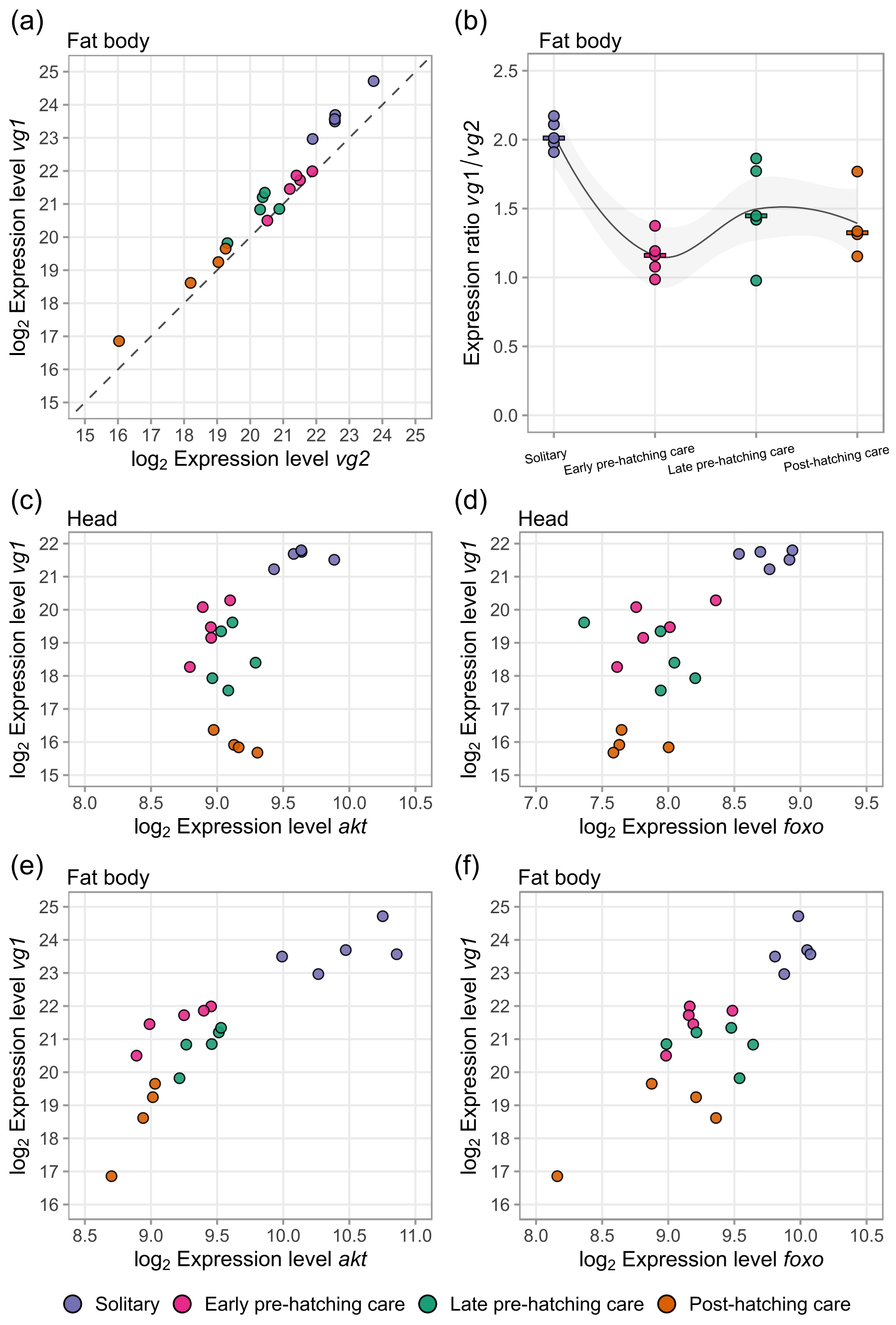

Figure S9: The expression of *vg2 (vitellogenin 2*, LOC108565867) and *vg1* (*vitellogenin 1*, LOC108564206) was highly correlated in the fat body (a; negative binomial GLM LRT χ^2 (1) = 641.55, p < 0.001; the dashed line indicates equal expression levels of *vg1* and *vg2*; note that axes are log-scaled). In the fat body, the gene expression ratio between *vg1* and *vg2* in the brood care phases was roughly half of that in the solitary phase (b; expression ratio *(vg1/vg2)* ~ phase; GLM LRT $F_{3,15}$ = 12.42, p < 0.001). Expression of *akt* (c, e; protein kinase B; LOC108559424) and *foxo* (d, f; forkhead box O; LOC10856804) significantly explained variation in *vg1* expression in both tissues (negative binominal GLM; ANOVA LRT all p < 0.001). Dots represent individual samples and lines (LOESS) show expression pattern with shaded areas indicating the 95% confidence interval. Expression levels in (a) and (c - f) are plotted as log_2 transformed normalized counts. Horizontal bar shows median of samples per phase and tissue (b).

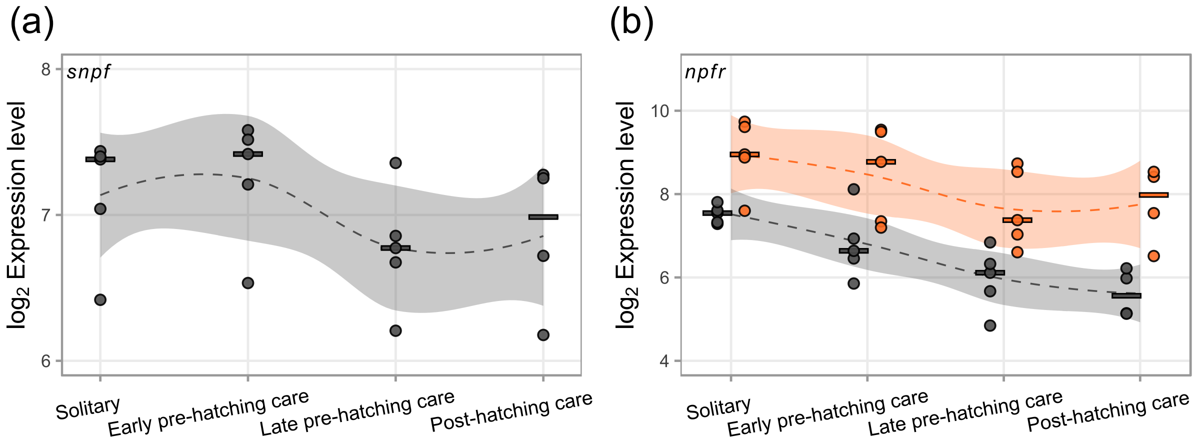

Figure S10: The expression levels of *short neuropeptide f* (a; *snpf* LOC108562743) decreased in the fat body from the solitary to the post-hatching care phase (LRT-p_{adj} < 0.001). The gene was not expressed in the fat body. Expression of its receptor (b; *npfr* LOC108564901) decreased in the head (grey, p_{adj} < 0.001) while there was no robust change in the fat body (orange; p_{adj} = 0.12). Expression levels are plotted as log_2 transformed normalized counts. Dots represent individual samples and dashed lines (LOESS) show expression pattern with shaded areas indicating the 95% confidence interval. Horizontal bar shows median of samples per phase and tissue.

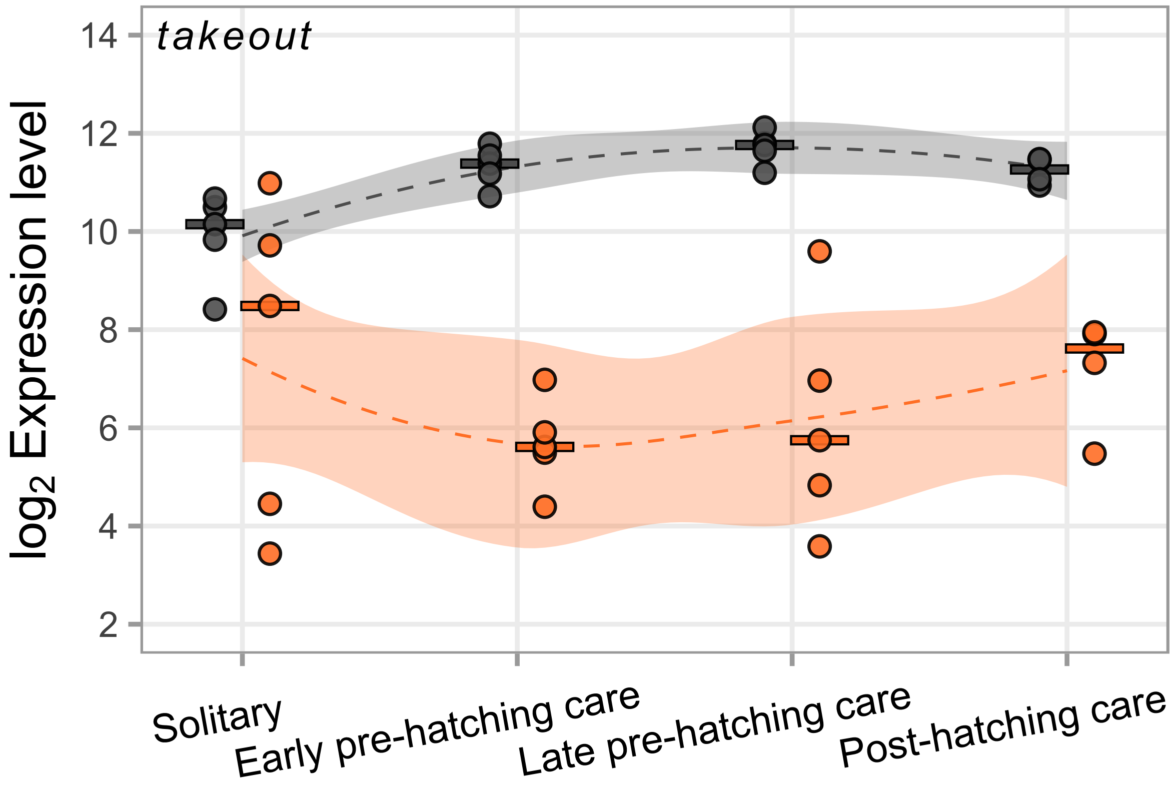

Figure S11: Expression levels of *takeout* (LOC108560804) in the head and fat body across a solitary phase and three parental care phases. In the head, *takeout* expression increased after the beetles moved onto a carcass, with the highest expression during the late pre-hatching care phase (grey; LRT-$p_{adj} < 0.001$). In the fat body, expression declined with onset of parental care and reached its lowest level during the early pre-hatching care phase (orange; Fat body: $p_{adj} < 0.05$). Expression levels are plotted as $\log_2$ transformed normalized counts. Dots represent individual samples and dashed lines (LOESS) show expression pattern with shaded areas indicating the 95% confidence interval. Horizontal bar shows median of samples per phase and tissue.

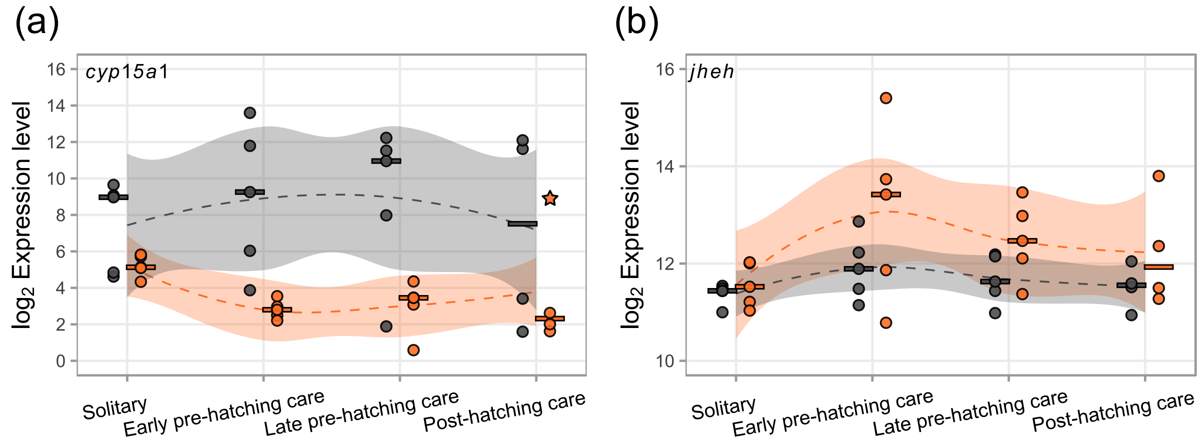

Figure S12: Expression levels of *cyp15a1* (a; *methyl farnesoate epoxidase*; LOC108561482) and *jheh* (b; *jh epoxide hydrolase*; LOC108561631) involved in JH biosynthesis and degradation in the head and fat body across a solitary phase and three parental care phases. Head (grey) expression of both *cyp15a1* (LRT-p_{adj} = 0.59) and *jheh* (p_{adj} = 0.32) remained stable across phases, whereas expression in the fat body (orange) decreased significantly across phases (*cyp15a1* $p_{adj} < 0.001$; star indicates the removed fat body outlier; see methods); *jheh* $p_{adj} < 0.05$). Expression levels are plotted as $\log_2$ transformed normalized counts. Dots represent individual samples and dashed lines (LOESS) show expression pattern with shaded areas indicating the 95% confidence interval. Horizontal bar shows median of samples per phase and tissue.

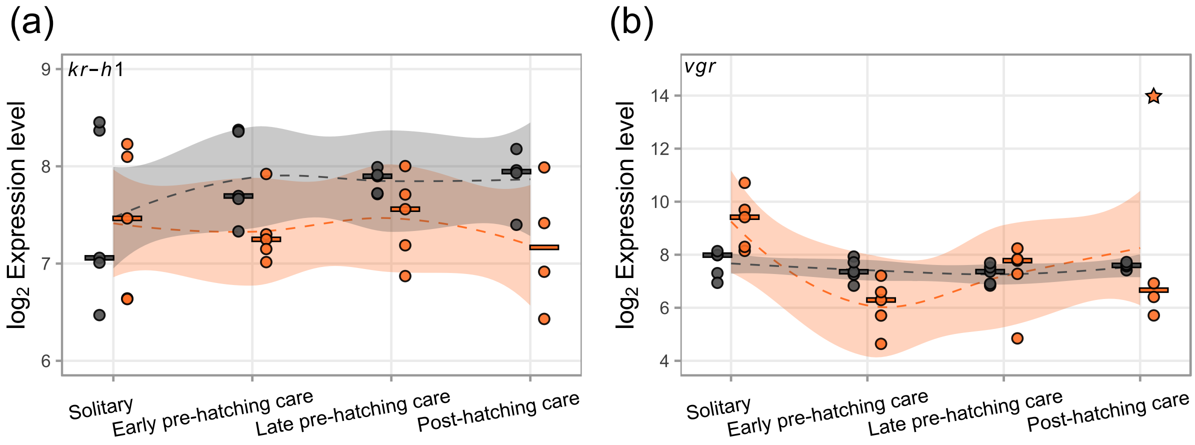

Figure S13: Expression levels of *krüppel-homolog 1 (a; kr-h1;* LOC108562898*)* and *vitellogenin receptor* (b; *vgr;* LOC108558983) in the head (grey) and fat body (orange) across a solitary phase and three parental care phases. *kr-h1* expression remained stable across phases in both tissues (Head: LRT-p_{adj} = 0.95; Fat body: p_{adj} = 0.89). *Vgr* head expression remained stable, but changed across phases in the fat body, with the highest expression during the solitary phase (Head: p_{adj} = 0.58; Fat body: p_{adj} < 0.001; star indicates the removed fat body outlier; see methods). Expression levels are plotted as log_2 transformed normalized counts. Dots represent individual samples and dashed lines (LOESS) show expression pattern with shaded areas indicating the 95% confidence interval. Horizontal bar shows median of samples per phase and tissue.
